# Cell Surface Deazaflavin-Diazirine Energy-Transfer (DarT) Photoproximity Labeling Using Antibody Conjugates

**DOI:** 10.1021/acs.bioconjchem.6c00247

**Published:** 2026-08-07

**Authors:** Judith M. Müchler, Leander B. Crocker, Max Ruwolt, Kristin Kemnitz-Hassanin, Christian P. R. Hackenberger

**Affiliations:** † Leibniz-Forschungsinstitut für Molekulare Pharmakologie (FMP), Robert-Rössle-Strasse 10, 13125 Berlin, Germany; ‡ Department of Chemistry, Humboldt Universität zu Berlin, Brook-Taylor-Straße 2, 12489 Berlin, Germany; § Institute of Chemistry and Biochemistry, Freie Universität Berlin, Thielallee 63, 14195 Berlin, Germany

## Abstract

Elucidating protein–protein interactions plays a crucial part in understanding disease mechanisms and advancing pharmacological research. Photocatalytic proximity labeling using antibody–catalyst conjugates enables the highly target-specific analysis of protein–protein interactions on the cell surface without altering the native cellular state through genetic manipulation. Here, we describe an extension of the deazaflavin–diazirine energy-transfer (DarT) labeling platform through the development and evaluation of trastuzumab–deazaflavin (Tra–dFl) conjugates for mapping the extracellular microenvironment of human epidermal growth factor receptor 2 (HER2). Four Tra–dFl conjugates were synthesized via azide–DBCO click chemistry, varying in PEG linker size and catalyst loading: Tra–PEG0–dFl, Tra–PEG6–dFl, Tra–PEG12–dFl, and Tra-bis-dFl, exhibiting a branched linker for dual attachment. Imaging and proteomic pulldown experiments revealed that linker size influences biotinylation efficiency and proteomic enrichment, resulting in Tra–PEG12–dFl emerging as the most effective construct, enabling the enrichment of cancer-associated cell surface proteins in HER2-positive SK-BR-3 cells. Evaluation of catalyst valency using a branched linker resulted in fewer enriched proteins, suggesting that linker architecture is a more critical parameter for conjugate performance than increased catalyst loading. Together, these findings provide guidelines for antibody-based deazaflavin conjugates and expand the applicability of DarT labeling for target-directed surfaceome mapping.

## Graphical abstract

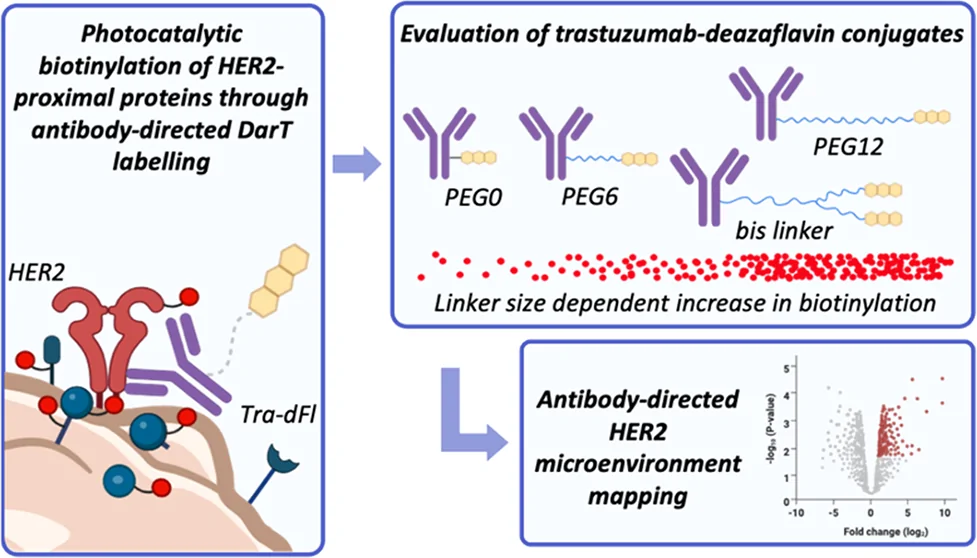

## Introduction

Proximity labeling has been the method of choice to study dynamic, extracellular protein–protein interactions (PPIs) for more than two decades, and recent advances in the different labeling techniques have elevated the performance and practicality immensely.^1,2^ In principle, the goal of proximity labeling is to target a protein of interest (POI) in its native cellular state and covalently tag nearby proteins with enrichment or purification tags by locally activating an otherwise inert species to allow subsequent separation and identification of tagged proteins. Originally, enzymatic approaches were used for proximity labeling; however, recent advances in photocatalytic strategies have shown great potential for affinity-directed labeling of a POI’s environment with high spatial resolution (Figure 1A).^3^ For example, photoproximity labeling (PPL) can help to elucidate PPIs within the cell surface proteome (or surfaceome), a critical interface between intra- and extracellular environments that affects fundamental processes such as signal transduction or intercellular communications. Importantly, the surfaceome is highly dynamic: upon internal or external stimuli, membrane proteins can dimerize or rearrange into multimers, even clusters in the micrometer range.^4^ Consequently, when studying extracellular disease markers, considering the specific cellular environment can provide essential insights into the mechanistic details of disease progression and perhaps identify new drug targets.

**Figure 1:**
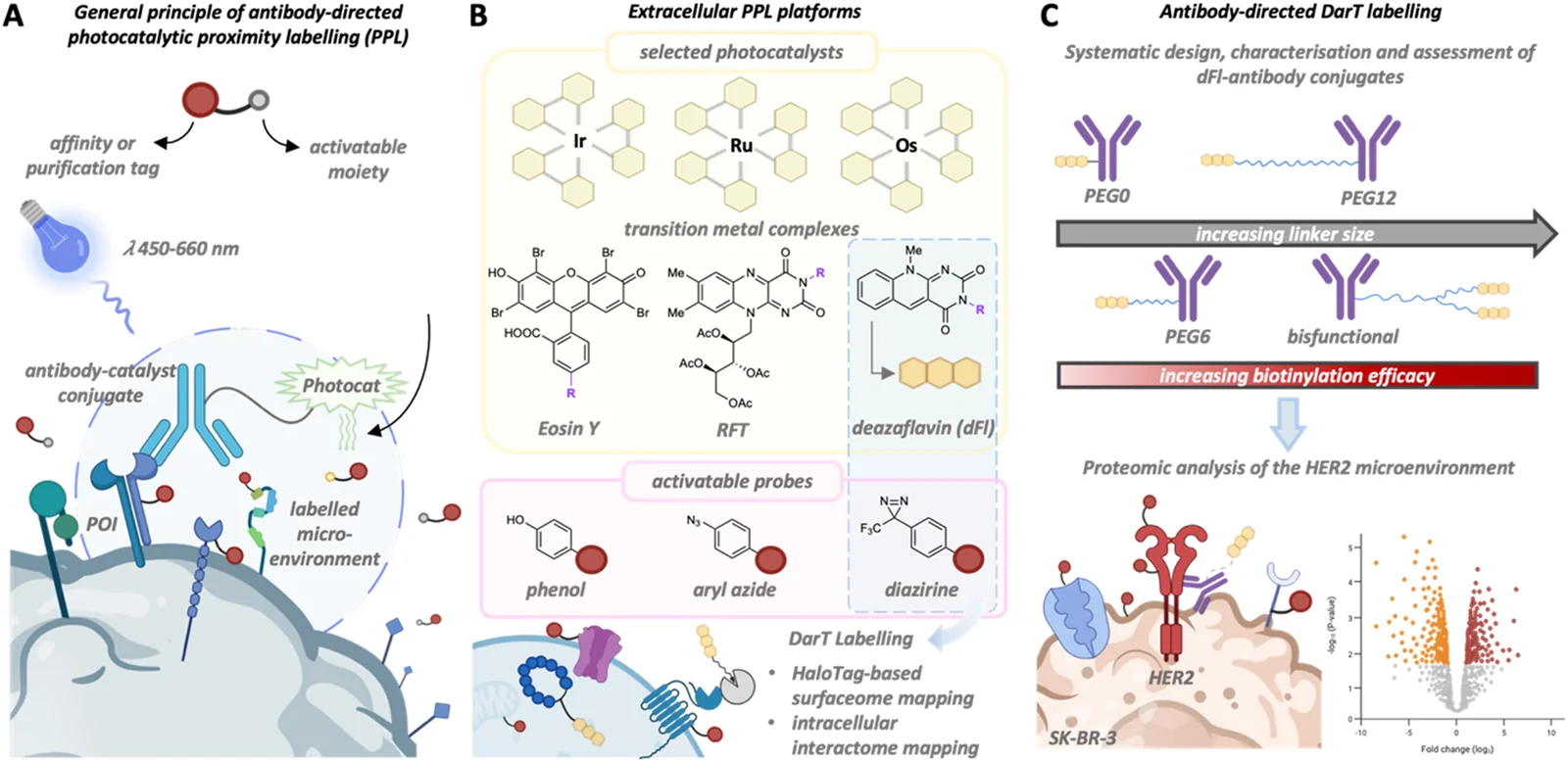
(A) Schematic of the general principle behind photocatalytic proximity labeling: the locally restricted activation of a biotin moiety through antibody targeting upon irradiation with visible light. (B) Depiction of selected photocatalysts and activatable probes that have been published for extracellular labeling, including the DarT platform consisting of deazaflavin and diazirine-biotin. (C) The scope of this work, demonstrating the applicability of DarT labeling for extracellular antibody-targeted PPL through the investigation of different antibody conjugates. This figure (excluding chemical structures) was created with BioRender.com.

The combination of antibodies as targeting tools together with visible light photocatalysis for PPL of cell surface targets has led to enhanced spatiotemporal resolution of the labeling reaction and highly reliable PPI elucidation. For example, the programmed death-ligand 1 (PD-L1) microenvironment has been investigated with unmatched precision using an iridium (Ir) photocatalyst for diazirine activation to generate highly reactive carbenes.^5^ Besides diazirines, aryl azides and phenols have emerged as commonly applied labeling probes in combination with a number of photocatalysts (Figure 1B).^6^ For example, the combination of osmium-based catalysts together with aryl azides was used to form nitrenes upon red light irradiation in order to map the microenvironment of epithelial cell adhesion molecule (EpCAM).^7^ While highly efficient, transition-metal complexes also connote intrinsic cytotoxicity^8^ and lengthy syntheses,^5,7,9^ which have inspired the exploration of more readily available and biocompatible alternatives.

Organic photosensitizers have quickly been identified as auspicious alternatives. Commercially available dyes of the triarylmethane scaffolds (*e.g*., thiorhodamine^10^ or dibromofluorescein^11,12^ ) have been used to locally generate singlet oxygen for subsequent reaction of biotin-hydrazide or amines with oxidized histidine residues. The discovery of Eosin Y as a multiscale photocatalyst furthermore demonstrated broader applicability through its ability to activate different probes for tunable resolution of the PPL reaction.^13^

Another group of organic compounds capable of photo­(redox) catalysis is flavins: different flavin derivatives have been used in PPL, primarily for efficient single electron transfer to phenols or singlet oxygen generation.^14–17^ Recently, our lab has identified and characterized deazaflavin (dFl) as a potent photocatalyst additionally capable of energy transfer to diazirines and aryl azides, therefore extending the toolbox of flavin-based PPL. We regard the deazaflavin–diazirine energy-transfer (DarT) strategy as especially valuable due to its high-resolution, low background labeling, and could demonstrate extracellular and intracellular PPL, the latter over a period as long as 24 h due to the excellent biocompatibility of dFl (Figure 1B, bottom).^18^

Previously, we demonstrated DarT labeling for cell surfaceome mapping by utilizing a HaloTag fusion protein that relies on cellular transfection of genetic material. However, transfection often has variable efficiency and the HaloTag construct can potentially alter the cell’s native structure or function due to the presence of an unnaturally fused enzyme. The use of antibody-photocatalyst conjugates for PPL offers high target specificity without the need for genetic manipulation, with a large number of commercially available antibodies permitting high accessibility to researchers across scientific disciplines. While our earlier work introduced two antibody-dFl conjugates capable of cell surface biotinylation, their application to surfaceome mapping was not yet demonstrated. In this current work, we optimized dFl-antibody conjugates for extracellular DarT labeling and chose human epidermal growth factor receptor 2 (HER2) as our model target with trastuzumab (Tra) as the targeting antibody. HER2 is an oncogenic protein overexpressed in 20–30% of breast cancers amid other cancer types, allowing tumors to proliferate more aggressively but also enabling targeted therapies with monoclonal antibodies trastuzumab and pertuzumab.^19^ We show a linker size-dependent influence of the biotinylation efficiency of Tra-dFl conjugates, a factor that, to our knowledge, has not been assessed previously for PPL reactions. The strategic investigation of linker size and catalyst loading resulted in the successful pulldown and proteomic analysis of the HER2 microenvironment of SK-BR-3 cells, thereby offering a strategic guide to antibody-based DarT labeling (Figure 1C).

## Results

### Design and Synthesis of dFl-Conjugated Antibodies

Previous work on our DarT labeling platform has introduced two antibody-dFl conjugates enabling cell surface biotinylation, confirmed through live-cell imaging.^18^ One conjugate consisted of Tra functionalized with a dFl-ethynyl-triazolyl-phosphinate derivative (Tra-ETP-PEG6-dFl), selectively reacting with cysteine residues;^20^ the other was a lysine-modified secondary IgG, prepared analogously to other flavin-based PPL techniques (Figure 2A).^14^ However, when these conjugates were used for PPL followed by proteomic analysis, the resulting pulldown samples did not allow a meaningful interpretation in the biological context of HER2 (Figure S1). Since we included many variables in our first set of antibody conjugates (monoclonal primary *vs* polyclonal secondary antibody, conjugation strategy, and overall linker design), we decided to follow a more systematic approach to optimize antibody-dFl conjugates for their applicability in proteomic pulldowns. Our goal was to increase the degree of biotinylation on proteins surrounding our target using Tra-dFl conjugates to achieve a robust pulldown of biotinylated proteins. The effect of conjugate design on its ability to biotinylate the cell surface can be observed by live-cell imaging, while mass spectrometry-based proteomics following a biotin pulldown can give a more profound insight into the suitability of the respective antibody conjugate by identifying isolated proteins. Therefore, we investigated the effect of varying linker lengths and dFl valency on Tra facilitated through NHS-ester-mediated lysine modification using confocal microscopy and mass spectrometry (MS).

**Figure 2:**
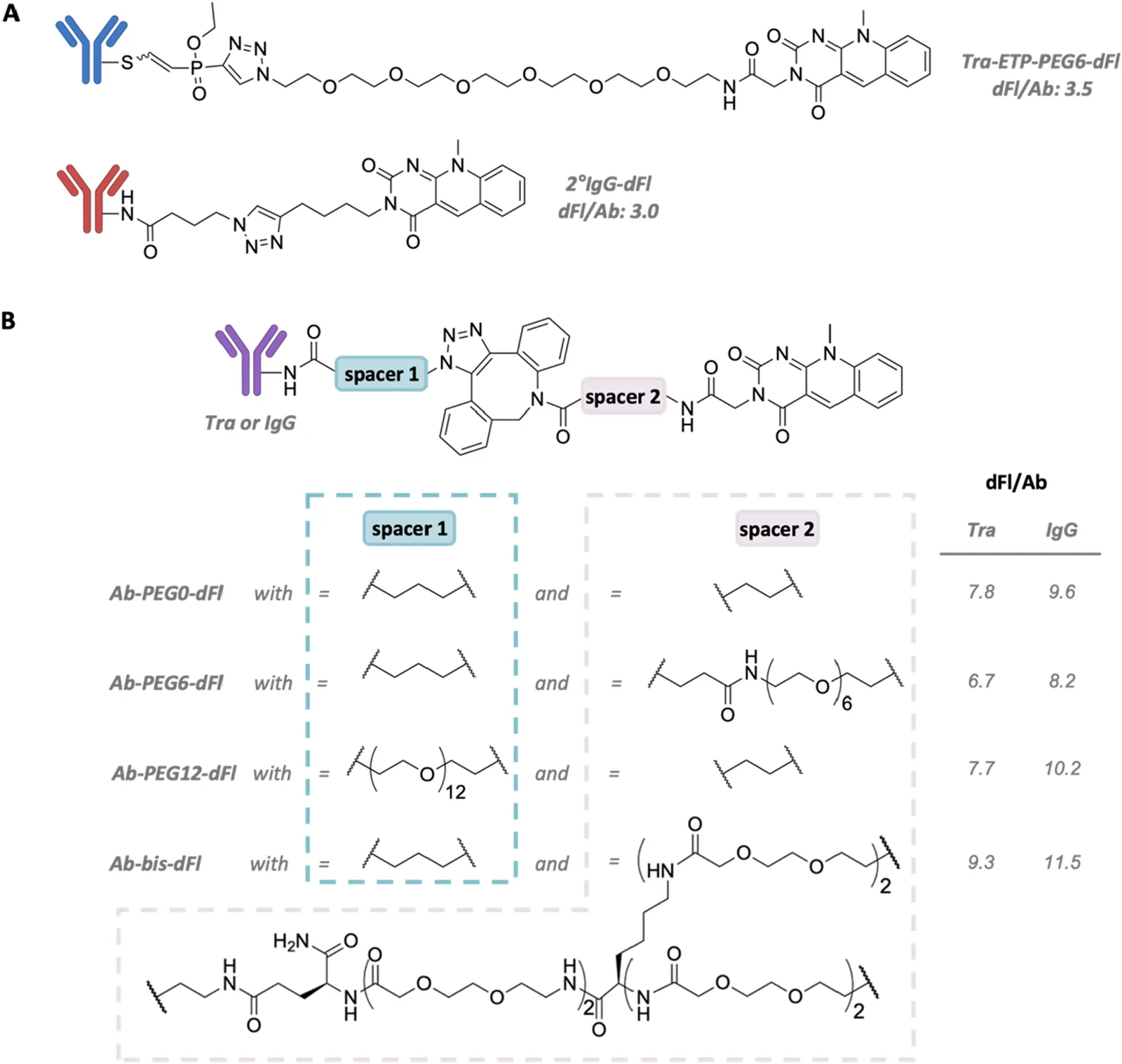
(A) First set of antibody-dfl conjugates used in previous studies. (B) General design of antibody-dFl conjugates generated via SPAAC, achieving conjugates of different linker sizes (PEG0-PEG12) as well as a bis-functional conjugate. A detailed preparation scheme can be found in the Supporting Information (Figure S2) with indication of the specific azide and DBCO compounds used for each conjugate.

We hypothesized that the intrinsic hydrophobicity and comparably small size of the dFl catalyst would require the implementation of a hydrophilic linker into the antibody-dFl conjugate. Inserting a polyethylene glycol (PEG) spacer between the antibody and dFl might lead to better exposure of dFl for energy transfer to diazirine-biotin and therefore to higher biotinylation efficiency. PEG spacers are commonly used to counteract hydrophobic moieties in antibody-drug conjugates (ADCs), reduce aggregation, and achieve higher conjugation ratios.^21,22^ Since we were curious to explore how different linker sizes will affect biotinylation, this approach conveniently allows for linker length variation simply by using commercially available, bifunctional PEG linkers with a variable number of PEG units. It has been shown for the Tra-HER2 system that if the distance between catalyst and target is varied by site-specific implementation of the catalyst onto the targeting moiety (in this case: Fab of trastuzumab), it affects the labeling pattern *in vitro* as well as on the surface of SK-BR-3 cells.^23^ We therefore assumed that varying the leash length between catalyst and antibody by implementing different amounts of PEG units will likewise influence the labeling reaction.

For antibody conjugation, the well-established monoclonal antibody trastuzumab (Tra) was selected as a HER2-targeting moiety, while a polyclonal anti-mouse IgG was used as a control for unspecific binding to account for background biotinylation during blue light irradiation. Antibodies were modified by installing azide handles onto lysine residues using NHS esters to allow strain-promoted azide–alkyne cycloaddition (SPAAC) with DBCO-dFl compounds.^24–26^ Instead of using thiols as before, we chose to functionalize amine residues to achieve a higher degree of modification due to the availability of ubiquitous conjugation sites. Additionally, the combination of NHS-azide and DBCO-catalyst compounds has recently been demonstrated to result in a higher degree of biotinylation in *in vitro* protein labeling reactions than other conjugation strategies.^13^ Owing to the modularity of the SPAAC reaction, antibody-dFl conjugates were effectively generated by combining NHS–azide derivatives of different lengths with DBCO–dFl compounds that either contained or lacked a PEG spacer (Figure S2). In this manner, we prepared antibody-dFl variants of varying linker: long (Ab-PEG12-dFl), medium (Ab-PEG6-dFl), and short (Ab-PEG0-dFl; Figure 2B). Since we used lysine-directed modification of the antibodies with NHS esters, we assessed the catalyst distribution of those three Tra-dFl conjugates by recording intact protein mass spectra of the modified antibodies. Although this method does not allow to allocate the specific modification sites, we observed a comparable distribution of catalyst over the light and heavy chains of the antibody for all three conjugates (Figure S3). Therefore, we assume similar catalyst loading for all antibody-dFl conjugates applied in this study.

Previously, we observed a lower degree of diazirine-mediated photolabeling using dFl relative to state-of-the-art Ir catalysts, likely contributing to fewer proteomic hits obtained with dFl in parallel HaloTag-based PPL experiments.^18^ We therefore wondered if an increase in dFl valency per antibody could reinforce the labeling ability of antibody-dFl conjugates. Hence, we envisioned the synthesis of a bis-functional dFl linker on solid support that is compatible with solid-phase peptide synthesis (SPPS). After being equipped with a DBCO moiety, the DBCO-bis-dFl probe is suitable for SPAAC (Figure 2B). This linker construct contains eight PEG units, but additional amide bonds and a CH_2_ spacer result in an even higher distance between the catalyst and antibody compared to the PEG12 linker. With these conjugates, we had several antibody conjugates in hand that allowed us to additionally investigate the effect of an increased dFl valency. Because dFl possesses a distinct absorption maximum at 391 nm, the prepared antibody-dFl conjugates were characterized by absorption measurements to determine protein and dFl molar concentrations and calculate the dFl-to antibody ratio (see Experimental Section and Figure S4).^18^ The conjugates with increasing PEG spacer yielded similar ratios of 6.7–7.8 dFl per Tra, once more suggesting comparable catalyst distribution among those conjugates and distinctly exceeding the number of conjugated dFl of both antibodies previously used (Figure 2A,B). The bis-linker approach indeed yielded a slightly higher dFl-to-antibody ratio of 9.3 for trastuzumab.

### Comparing Cell-Surface DarT Labeling via Confocal Microscopy

After preparation of the antibody conjugates, we assessed their capability to execute DarT labeling on live cells using confocal fluorescence microscopy. Fluorescence imaging of the biotinylation pattern on cells subjected to proximity labeling allows us to evaluate the labeling protocol with respect to antibody binding and the photolabeling reaction in a cellular context.^14,27^ To perform DarT labeling on the cell surface, SK-BR-3 cells, a breast cancer cell line that overexpresses HER2, were incubated with either Tra-dFl or polyclonal IgG-dFl conjugates that do not bind to a specific extracellular target on human cells. After the addition of diazirine-biotin, biotinylation is triggered by irradiation with 450 nm and visualized subsequently through incubation with a streptavidin dye (Figure 3A). After costaining the nuclei with Hoechst stain, cells were visualized by confocal fluorescence microscopy. Qualitative comparison of the acquired images reveals clear streptavidin staining on cells treated with Tra-dFl conjugates containing PEG6, PEG12, or the bis-dFl linker, whereas cells incubated with the short Tra-PEG0-dFl construct showed a noticeably weaker signal. At all conditions, the streptavidin stain is localized on the cell membrane, encasing the nucleus of each cell but lacking a dispersed intracellular signal (Figure 3B). Incubation with IgG-dFl derivatives or PBS prior to diazirine addition and irradiation did not lead to cell surface biotinylation (Figure 3C), showcasing the suitability of the polyclonal IgG as a nonbinding control. These qualitative observations were affirmed by quantifying and comparing the streptavidin signals of all conditions (Figure 3D). For that purpose, the mean integrated densities of the streptavidin channel from five images per condition were plotted against each other, after subtracting the background signal obtained from a sample treated with only PBS. The quantification helps to emphasize the difference between Tra and IgG treatment as well as the drastic increase in streptavidin signal upon the installation of a PEG spacer between the catalyst and antibody. The results we obtained from this microscopy readout suggest that employing a PEG linker into the conjugate, separating the catalyst from the antibody, provides better conditions for DarT labeling. Because microscopy can only deliver qualitative proof that biotinylation occurs in a cellular environment during a PPL experiment, without revealing which biomolecules are modified, we next applied our antibody platform to affinity-purification mass spectrometric analyses to isolate and identify biotinylated proteins.

**Figure 3:**
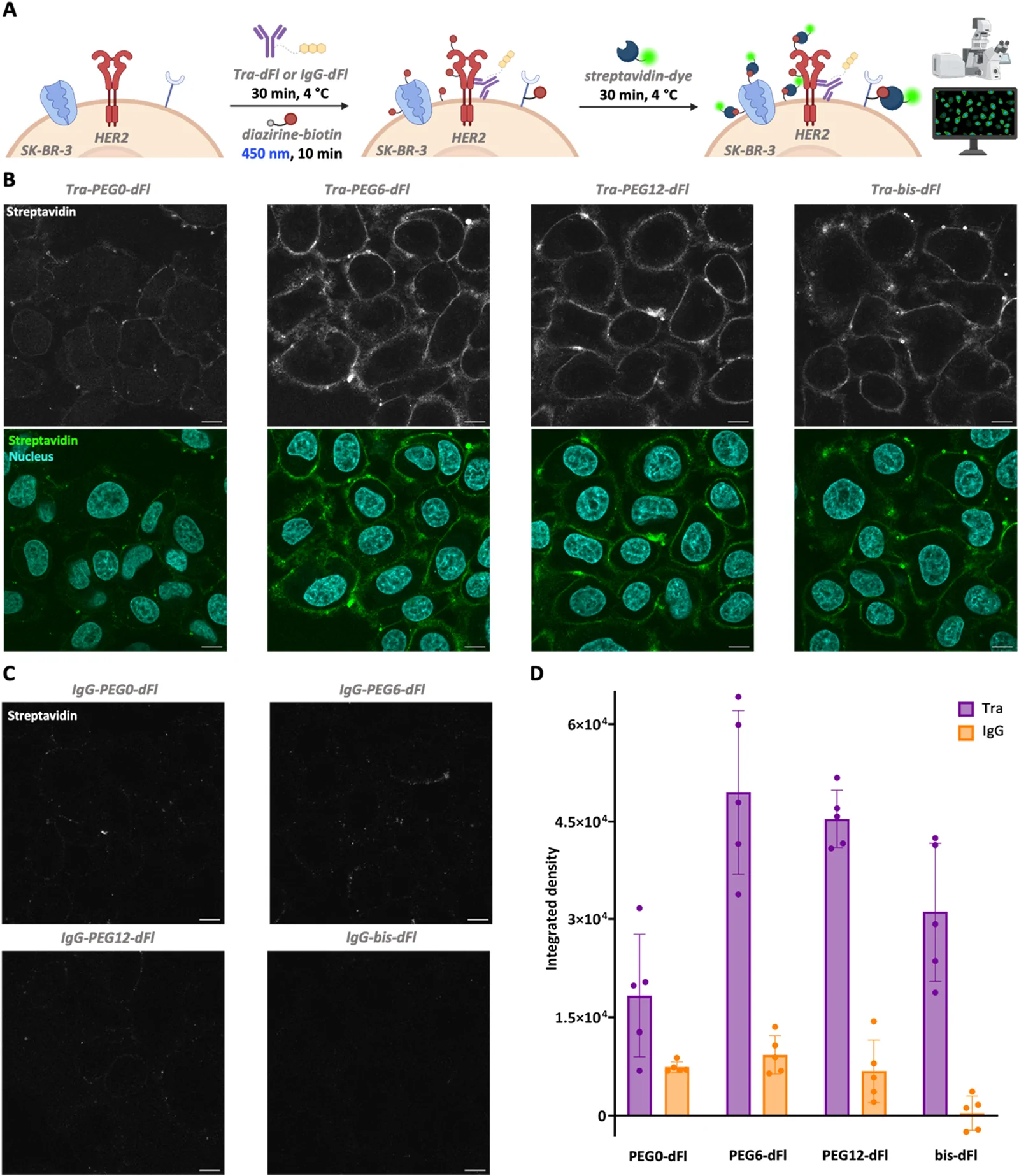
(A) Workflow for the assessment of biotinylation by microscopy using antibody-based, extracellular targeted DarT labeling on living cells. (B) Representative confocal microscopy images of SK-BR-3 cells treated with Tra-dFl conjugates. Biotinylation patterns are revealed by staining with Streptavidin-Alexa Fluor 488 (white and green), and Hoechst dye was used to define nuclei (cyan). Scale bar: 10 μm. (C) Representative confocal microscopy images of SK-BR-3 cells treated with IgG-dFl conjugates (nonbinding control). Biotinylation patterns are revealed by staining with Streptavidin-Alexa Fluor 488 (white). (D) Bar chart showing the quantified mean streptavidin signal of samples treated with Tra conjugates (purple) and IgG conjugates (orange). Individual data points are shown with dots and error bars represent the standard deviation of five replicates. Panel (A) was created with BioRender.com.

### Proteomic Investigation of the HER2 Microenvironment with DarT Labeling

To trial the applicability of our antibody-based DarT labeling approach for surfaceome mapping, we performed PPL on SK-BR-3 cells in a manner analogous to the imaging experiment, followed by proteomic analysis (Figure 4A). We selected those antibody conjugates that revealed promising biotinylation efficiency during microscopy, specifically those conjugates containing a PEG spacer (Figure 3B). Since IgG-dFl conjugates have been shown to be an appropriate control to depict targeting *vs* nontargeting conditions in tandem with Tra conjugates, we compared labeling of the HER2 microenvironment in the presence and absence of a targeting moiety. After labeling, cells were submitted to lysis and biotinylated proteins were enriched using magnetic streptavidin beads, followed by tryptic digest. Enriched proteins were identified by matching LC-MS/MS-detected peptide sequences to the human proteome.

**Figure 4:**
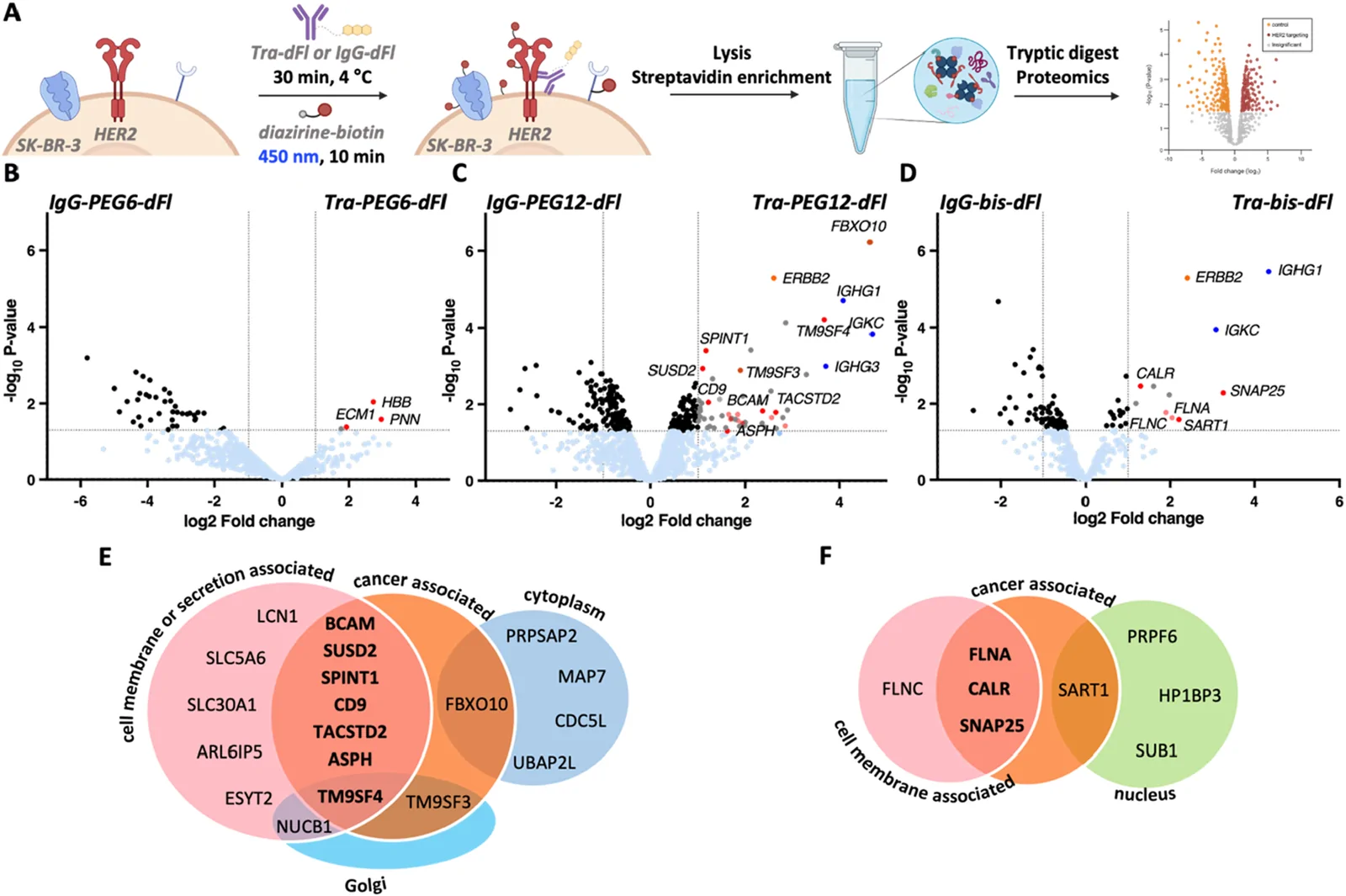
(A) Proteomic workflow for the identification of proteins biotinylated upon HER2-targeted DarT labeling using Tra- and IgG-dFl conjugates. (B–D) Volcano plots from streptavidin-enriched protein samples derived from cells treated with Tra-PEG6-dFl, Tra-PEG12-dFl, Tra-bis-dFl, or their respective nonbinding IgG-dFl control. Significantly enriched proteins (*P* < 0.05; log_2_(Fold change) > 1) are colored according to their respective biological relevance in this experiment: • orange ERBB2 (target) • blue IgG fragments (targeting moiety) • red associated with the cell membrane or periphery with reported links to cancer • light red associated with the cell membrane or periphery *without* reported links to cancer • ochre linked to cancer but associated with other cellular compartments. (E) Venn diagram displaying overlap between significantly enriched proteins derived from Tra-PEG12-dFl treatment with reported connections to cancer mechanisms and their respective cellular localization. (F) Venn diagram displaying overlap between significantly enriched proteins derived from Tra-bis-dFl treatment with reported connections to cancer mechanisms and their respective cellular localization. Panel (A) was created with BioRender.com.

Despite showing promising cell surface labeling by microscopy, the Tra-PEG6-dFl conjugate did not yield significant enrichment of either HER2 (gene name: ERBB2) or antibody units. Self-labeling and pulldown of both the target and the targeting moiety serve as a strong indicator of successful PPL and enrichment, whereas the absence of such self-labeling indicates an unsuccessful experiment. Among the proteins that are enriched in Tra-PEG6-dFl-treated conditions are HBB and ECM1, which are located in the extracellular space and exhibit a connection to the progression of breast cancer (Figure 4B).^28,29^ Another protein enriched in this data set is pinin (PNN), part of the desmosome and associated with different cancer types, among others, through its role in activating the EGFR/ERK signaling pathway.^30–32^ However, the low number of enriched hits as well as the absence of self-labeled proteins challenge the suitability of the PEG6 conjugate for surfaceome mapping.

To explore the discrepancy of Tra-PEG6-dFl performance between the microscopy images and proteomic analysis, we decided to benchmark our conjugates and workflows against a Tra conjugate carrying the state-of-the-art Ir catalyst commonly used in extracellular PPL experiments.^5,23,33^ A Tra-PEG6-Ir conjugate matching the linker design of Tra-PEG6-dFl enables a direct comparison of the two catalysts, allowing us to attribute differences in cell surface biotinylation to catalyst-specific properties such as photocatalytic efficacy, size, and charge state (Figure S5A). Imaging cell surface biotinylation generated by Tra-PEG6-Ir reveals a profoundly more intense signal when compared with the dFl conjugate (Figure S5B). This difference aligns with previous benchmarking experiments comparing dFl and Ir, where the higher photocatalytic efficacy of Ir resulted in higher degrees of labeling in both *in vitro* BSA and HaloTag-mediated cell surface biotinylation.^18^ After submitting the antibody-Ir conjugates to our enrichment workflow followed by proteomic analysis, we can observe significantly enriched ERBB2 among a total of 84 enriched proteins in the Tra-PEG6-Ir-treated data set (Figure S5C). Comparison with the Tra-PEG6-Ir reference data suggests that the dFl catalyst in Tra-PEG6-dFl does not generate sufficient biotinylation to pass the threshold required for a pulldown and MS-based protein identification.

We next investigated conjugates with a longer linker structure, Tra-PEG12-dFl and Tra-bis-dFl, that yielded strong enrichment of ERBB2 as well as Ig fragments (IGHG1, IGHG3, and IGKC; Figure 4C,D) over nontargeting conditions, demonstrating successful application of HER2-targeted DarT labeling in a proteomic workflow. Upon Tra-PEG12-dFl treatment, 7 out of 13 enriched proteins directly associated with the cell membrane or periphery exhibit reported links to cancer: BCAM, SUSD2, SPINT1, CD9, TACSTD2, ASPH, and TM9SF4 (Figure 4C,E). We found that 3 out of those 7 proteins, SUSD2, SPINT1, and CD9, are significantly enriched in the Tra-PEG6-Ir treatment data set as well. The enrichment of cell adhesion promoters BCAM and SUSD2 from a HER2-targeting PPL experiment is in accordance with a study currently available as a preprint utilizing Tra-Ir conjugates across 11 cell lines, listing BCAM and SUSD2 among the 12 proteins most commonly enriched in HER2-positive cell lines.^34^ A different study also identifies BCAM among the most confident protein hits upon treatment with diverse Tra Fab-Ir conjugates that were applied to SK-BR-3 cells.^23^ SPINT1 is a hepatocyte growth factor activator inhibitor reported to inhibit cell surface proteases in breast cancer cells and thus potentially contributes to cancer cell survival.^35^ The protein interestingly shows an association with HER2-positive breast cancer through its elevated expression in this subtype.^36^ TACSTD2 encodes for TROP2, the target of ADC hIMB1636-LDP-AE, and elevated expression levels have been connected to high-expressing HER2 tumors in transitional cell carcinoma.^37^ Enrichment of TACSTD2 in our data may raise the possibility of a connection between HER2 and TROP2 in breast cancer as well. Moreover, TM9SF4 and TM9SF3, both members of the transmembrane 9 superfamily, have been shown to be critically involved in tumor growth and proliferation.^38,39^ Although both proteins mainly reside in the Golgi apparatus, TM9SF4 has been reported to additionally localize on plasma and endosomal membrane.^40,41^ While ASPH is a well-known metastasis promoter and disease marker associated with HER2-amplified carcinoma,^42,43^ CD9 exhibits tumor-promoting as well as inhibiting properties.^44^ An additional six of the 49 enriched proteins are located at the plasma membrane or the extracellular space without current evidence for oncogenic links (Figure 4E).

While the analysis of our sample set treated with Tra-bis-dFl foremost revealed successful pulldown of HER2 and trastuzumab, the data set yields only 8 significantly enriched proteins in relation to its nontargeting IgG control, which is roughly 20% of proteins enriched by Tra-PEG12-dFl treatment with the same protocol. Proteins significantly enriched upon Tra-bis-dFl treatment demonstrate a notable overlap between membrane-associated or secretory proteins and proteins involved in cancer-associated mechanisms (FLNA, CALR, and SNAP25; Figure 4F).^45–47^ However, besides the target and targeting moiety, no significantly enriched proteins overlap between Tra-PEG12-dFl and Tra-bis-dFl data sets (Figure S6).

## Discussion

In this work, we investigated the biotinylation efficiency of an array of antibody-deazaflavin conjugates for cell-surface proteomic analysis using diazirine photolabeling probes. We observed that the incorporation of a PEG linker was crucial for achieving the biotinylation threshold required for proteomic applications. Visualizing cell surface biotinylation through microscopy distinctively revealed the increase in biotinylation for Tra-dFl conjugates containing a PEG spacer (Tra-PEG6-dFl, Tra-PEG12-dFl, and Tra-bis-dFl) in comparison to the Tra-PEG0-dFl conjugate. These data lead us to the assumption that a short and rigid linker, as used in Tra-PEG0-dFl, leads to insufficient solvent exposure of the catalyst, deeming it inaccessible for the activation of diazirine-biotin and therefore resulting in reduced cell surface biotinylation. Deazaflavins are commonly explored drug scaffolds binding several proteins through hydrophobic interactions of their tricyclic core, hydrogen bonding, or electrostatic interactions of heteroatoms.^48–50^ In the PEG0 conjugate, this propensity to interact with secondary and/or tertiary protein structures could lead to dFl being shielded by the antibody’s size or even buried within the antibody, prohibiting close contact to diazirines that is needed for energy transfer. The DBCO moiety of the DBCO-0-dFl derivative additionally contributes to the overall hydrophobicity of the conjugate (Figure 2A). When going a step further and applying the promising PEG containing conjugates to a proteomic pulldown of the HER2 environment on SK-BR-3 cells, we observed another restriction: only the two conjugates with a long PEG spacer, Tra-PEG12-dFl and Tra-bis-dFl, were able to achieve sufficient biotinylation of cell surface proteins, resulting in the enrichment of HER2 protein neighbors, with enrichment of self-labeled antibody and HER2 being an important indicator for this success, which was lacking in the Tra-PEG6-dFl-treated data set.

Despite the Tra-PEG-dFl conjugates showing promising biotinylation in the microscopy readout, microscopy is primarily useful in antibody-based PPL to confirm conjugate binding and subsequent labeling at the target site; it does not directly demonstrate functional interactome mapping. Cell surface biotinylation detected by microscopy cannot distinguish between labeling of membrane lipids, extracellular matrix components, or proteins. Consequently, protein identification approaches, such as MS-based proteomics, are required to assess potential protein interactors. While Tra-PEG6-dFl and Tra-PEG12-dFl generated visually similar biotinylation patterns, their distinct profiles of enriched proteins revealed differences likely connected to catalyst mobility. Moreover, pulldown data obtained through treatment with the analogous Tra-PEG6-Ir conjugate showed the successful enrichment of HER2 and proximal cell surface proteins. The differences in biotinylation observed between Tra-PEG6-dFl and Tra-PEG12-dFl, as well as between Tra-PEG6-dFl and Tra-PEG6-Ir, suggest that accessibility of the catalyst to the cellular environment is a limiting factor for reaching the photocatalytic efficiency required for antibody-directed protein labeling. This hypothesis is based on the observation that targeted proteomic readout was only possible when either the linker in the Tra-dFl conjugate was extended beyond PEG6 or dFl was replaced by the Ir catalyst. While the improved performance of Tra-PEG6-Ir is likely partly due to the generally higher efficiency of the Ir catalyst, its larger size and charged state may also contribute to better spatial separation from the antibody and thereby increase the catalyst exposure at the cell surface. We therefore assume that the PEG6 linker may be insufficient to fully expose dFl to perform efficient energy transfer catalysis, given that PEG linkers in an aqueous environment do not extend proportionally with increasing PEG units and are known to adopt folded conformations, particularly when flanked by hydrophobic moieties, as in the Tra-PEG6-dFl conjugate.^51,52^ This is coherent with previous experiments using our first Tra-dFl conjugate containing a PEG6 linker (Tra-ETP-PEG6-dFl, Figure 2A), which reportedly accomplishes visualizable cell surface biotinylation^18^ but failed to enable the pulldown of HER2-associated proteins (Figure S1).

Comparing proteomic data sets derived from Tra-PEG12-dFl and Tra-PEG6-Ir, we significantly enriched 13 (26%) cell membrane or extracellular located proteins through Tra-PEG12-dFl treatment and 14 (17%) through treatment with the Ir conjugate (Figure S5E,D). The appearance of SUSD2, SPINT1, and CD9 as significantly enriched in both sets strongly suggests close proximity of those proteins to HER2 on SK-BR-3 cells, a link that requires further validation and contextualization. Furthermore, Tra-PEG6-Ir enriched proteins with prominent roles in ferroptosis mechanisms (PRNP and ACSL4), which reportedly take place upon mitochondrial membrane collapse through radical oxygen species generated by excited Ir­(III) complexes (Figure S5D).^53–56^ No indicators of phototoxicity were enriched from dFl-treated cells.

Regarding the bifunctional Tra-bis-dFl conjugate, proteomic pulldown data revealed a relatively low number of cell membrane-associated hits as well as a lack of overlap in enriched proteins with the Tra-PEG12-dFl data set, which made us re-evaluate the structure of this bis-dFl conjugate. We assume that the size of the attachment, consisting of eight PEG units per branch and additional distance-generating connectors such as amide bonds or methylene groups, is the main contributor to sufficient dFl exposure necessary for robust photocatalytic activity. While the branched linker structure was intended to yield a higher dFl-to-antibody ratio, the proximity of two dFl molecules in one attachment might actually be counterproductive, as deazaflavins can undergo photodimerization upon irradiation, resulting in a loss of photocatalytic properties.^57,58^ Furthermore, the formation of noncovalent stacked dimeric complexes can lead to static self-quenching and ultimately to low-efficient energy transfer.^59,60^ The enrichment of HER2 and IgG fragments, together with only a limited number of additional significantly enriched proteins in the Tra-bis-dFl data set, suggests that the beneficial effect of sufficient catalyst mobility due to linker size may be counterbalanced by adverse reactions between catalyst molecules. If a branched linker is desired, these interactions are likely reduced if longer PEG spacers are introduced within the branches. However, our findings indicate that a single-chain dFl attachment with an appropriately sized PEG spacer represents a more favorable strategy toward antibody-mediated DarT labeling.

## Conclusion

With this work, we present an extension of the DarT labeling platform toward antibody-based PPL workflows. We systematically optimized antibody-dFl conjugates using trastuzumab as a proof-of-concept in order to investigate the protein environment of the cell surface receptor HER2. In our efforts to create a strategic labeling protocol, we tested four Tra-dFl conjugates consisting of similar azide-DBCO clicked core elements, but a varying number of PEG units in imaging as well as proteomic pulldown experiments. During imaging experiments, we observed that the biotinylation efficiency is influenced by linker size, declaring the short and rigid PEG0 linker as inadequate. A critical PEG spacer length was further confirmed by comparing PEG6 and PEG12 conjugates in our pulldown experiments, as the PEG6 conjugate did not enable successful pulldown of HER2-related proteins. Our Tra-PEG12-dFl conjugate therefore emerged as a useful prototype for surfaceome mapping, suggesting the proximity of HER2 to cancer-associated cell surface proteins SUSD2, SPINT1, CD9, Trop-2, TM9SF4, TM9SF3, ASPH, and BCAM. Except for SUSD2 and BCAM, these proteins have, to our knowledge, not yet been reported as potential HER2 interactors. As these conclusions are derived solely from proteomic enrichment data and PPL does not inherently discriminate between direct interactors and spatially proximal protein neighbors, further biological validation is required to confirm true interactions. Although we can report successful DarT labeling-mediated protein enrichment using a branched bis-dFl linker in which catalyst loading on the antibody was increased, a lower number of enriched proteins and the possibility of adverse interactions between two proximal dFl molecules lead us to the conclusion that the length of the linker is a more determining factor for suitable antibody-dFl conjugates than catalyst loading.

In summary, we present an important extension of the DarT labeling toolbox covering targeted surfaceome mapping that serves as a starting point for versatile directions in PPL applications. Our dFl-antibody conjugates are straightforward to prepare, relying on robust SPAAC reactions with a synthetically accessible dFl catalyst that exhibits a high biocompatibility. This biocompatibility will be particularly relevant for dFl-protein conjugates designed for intracellular applications or proteomic mapping over extended periods of time. In addition, understanding the influence of linker size on the effectiveness of a conjugate will help guide the development of further dFl-protein conjugates. In the future, we additionally plan to explore alternative photolabeling strategies, such as the use of cross-linking probes to unambiguously allocate protein–protein interactions, which have not yet been addressed by current PPL strategies.

## Experimental Section

### Preparation of Antibody-dFl Conjugates

The preparation of antibody-dFl conjugates is schematically displayed in Figure S2. The same protocol was applied for all conjugates. First, the azide and DBCO reagents were preincubated by mixing 4 μL of a 10 mM DMSO stock of the azide reagent with 16 μL of a 5 mM DMSO stock of the respective DBCO-dFl compound and leaving the reaction mixture in the dark for 1 h. The resulting 20 μL mixture was then added to 100 μL of a 2 mg/mL solution of either Trastuzumab or Goat anti-Mouse IgG in 100 mM carbonate-bicarbonate buffer (pH 9.2). The reaction was left to incubate overnight at 4 °C in the dark. Afterward, the solution was purified by two consecutive spin-filtration steps (Zeba Spin Desalting Columns, 40K MWCO, 0.5 mL) according to the manufacturer’s protocol. The degree of modification was determined by dividing the micromolar concentration of dFl (determined by absorbance measurement at 391 nm and comparison to a standard dilution of free DBCO-0-dFl) by the micromolar concentration of the antibody (determined through a bicinchoninic acid assay against a bovine serum albumin (BSA) standard dilution).

### HER2-Targeted PPL on Live Cells for Microscopy

To visualize surface biotinylation on live cells, 1.5 × 10^5^ cells per well were seeded in an eight-well glass-bottom slide (IBIDI) 1 day in advance. For each treatment or washing, the default volume used was 200 μL. Prior treatment, cells were washed with cold PBS and subsequently incubated with 3 μg Tra-PEG0-dFl, Tra-PEG6-dFl, Tra-PEG12-dFl, Tra-bis-dFl, Tra-PEG6-Ir, or the respective IgG-dFl conjugate as a control in DMEM growth medium F-12 with 5% fetal calf serum (FCS) for 30 min at 4 °C. Cells were then washed twice with FluoroBrite DMEM (Gibco) medium and treated with diazirine-biotin (250 μM) in FluoroBrite DMEM followed by irradiation with 450 nm light in a cold room (4 °C) for 10 min. After irradiation, cells were washed twice and incubated with streptavidin–Alexa Fluor 488 (3 μg; Invitrogen) for 30 min at room temperature. Cells were counterstained with 10 μg/mL Hoechst 33342 (Invitrogen) in FluoroBrite DMEM for 10 min and imaged in FluoroBrite DMEM. Imaging was performed using a Zeiss LSM710 confocal microscope with a x40, 1.4-NA Plan-Apochromat oil immersion objective at room temperature. Image analysis and processing were performed using FIJI software (version 2.16.0/1.54p)

### HER2-Targeted PPL on Live Cells for Proteomics

For the proteomic workflow, cells were seeded in 6 cm cell culture dishes in advance (n_Tra‑PEG6‑dFl/Ir_ = 2; n_Tra‑PEG12‑dFl/Tra‑bis‑dFl_ = 3). Three mL were used in each treatment step as well as for washing. At 80–90% confluency, cells were washed once in cold PBS before being treated with 5 μg of the respective antibody conjugate (Tra-PEG6-dFl, Tra-PEG12-dFl, Tra-bis-dFl, Tra-PEG6-Ir, or the corresponding IgG version). Cells were incubated for 30 min at 4 °C and afterward twice with cold PBS. Next, cells were brought into a solution of FluoroBrite DMEM containing 250 μM diazirine-biotin and irradiated with 450 nm light in a 4 °C cold room for 10 min. To harvest the cells, they were washed twice with PBS before 1 mL PBS was added to each dish and cells were scraped into 1.5 mL tubes precooled on ice and immediately processed further.

### Lysis and Enrichment

Harvested cells that underwent PPL treatment were pelleted by centrifugation (800 rpm, 4 °C, 4 min) and resuspended in 300 μL RIPA buffer containing protease inhibitors. Cells were lysed by sonication (3 min × 2 min, 30% amplitude), followed by debris centrifugation (16,000 rpm, 15 min, 4 °C). The supernatant was transferred to 1.5 mL protein low-binding tubes and protein concentration was determined through a bicinchoninic acid assay against a bovine serum albumin (BSA) standard dilution. For successful enrichment, at least 400 μg protein per sample is required. To enrich biotinylated proteins, a modified SP2E protocol was applied.^61^ A 1:1 mixture of 100 μL hydrophobic and hydrophilic carboxylate-coated magnetic beads was washed thrice with 500 μL water in 2 mL reaction tubes. Lysates (400 μg) were transferred directly onto the washed beads and ethanol was added to a final volume of 2 mL (final ethanol concentration must be >60% to ensure protein precipitation onto the beads). After resuspending the beads, the suspension was incubated for 5 min at RT and 950 rpm. Beads were washed three times with 80% ethanol in water (500 μL) using a magnetic rack, and proteins were eluted separately via the addition of 0.2% SDS in PBS (400 μL). This procedure was repeated twice, supernatants were combined and added to a 50 μL volume of equilibrated streptavidin-coated magnetic beads (three times prewashed with 0.2% SDS in PBS (500 μL)), followed by incubation for 2 h at RT in an end-over-end rotator for biotin–streptavidin binding. The beads were washed 3× with 0.1% NP-40 in PBS (500 μL), 3× with 6 M urea (500 μL), and 3× with water (500 μL). The washed bead mixtures were resuspended in 125 mM ammonium bicarbonate buffer (80 μL) and reduction and alkylation of the proteins were performed by the addition of 100 mM TCEP (10 μL) and 400 mM chloroacetamide (10 μL), followed by incubation for 5 min at 95 °C. Proteins were digested for 16 h at 37 °C with sequencing-grade trypsin (1.5 μL; 0.5 mg/mL). The supernatant containing the digested proteins was collected and the beads were subsequently washed three times with 30 μL 100 mM ammonium bicarbonate buffer. Before proceeding, a quality-control run was conducted using an XEVO G2-XS QToF instrument (Waters) to ensure complete digestion. The samples were then diluted based on this to ensure equal loading for subsequent LC-MS/MS measurements.

## Supplementary Material



## Data Availability

The mass spectrometry proteomics data have been deposited by the PRIDE partner repository and are available via the ProteomeXchange Consortium with the data set identifier PXD076830.

## Notes

Reagent and material index, description of applied methods and synthesis, as well as analytical data for all synthesized compounds (Figures S1–S5) (PDF)
