# Supplementary material for: Cell Surface Deazaflavin-Diazirine Energy-Transfer (DarT) Photoproximity Labeling Using Antibody Conjugates
Source: Bioconjug Chem. 2026 Aug 7;37(8):1678–88. doi: 10.1021/acs.bioconjchem.6c00247 (PMC13495547; doi:10.1021/acs.bioconjchem.6c00247)
Supplement: Supplementary file 1 [file bc6c00247_si_001.pdf]

Supporting Information

Cell Surface Deazaflavin-Diazirine Energy-Transfer (DarT) Photoproximity Labelling using Antibody Conjugates

Judith M. Müchler,[a,b] Leander B. Crocker,[a,*] Max Ruwolt,[c] Kristin Kemnitz-Hassanin,[a] Christian P. R. Hackenberger[a,b,*]

[a] Leibniz-Forschungsinstitut für Molekulare Pharmakologie (FMP), Robert-Rössle-Strasse 10, 13125 Berlin, Germany

[c] Institute of Chemistry and Biochemistry, Freie Universität Berlin, Thielallee 63, 14195 Berlin, Germany

[b] Department of Chemistry, Humboldt Universität zu Berlin, Brook-Taylor-Straße 2, 12489 Berlin, Germany

*corresponding author: crocker@fmp-berlin.de, hackenbe@fmp-berlin.de

Table of Contents

1. Supplementary Figures 1
2. Materials and Methods 7
  2.1 Reagents and Materials 7
  2.2 Methods 7
    Cell culture 7
    Characterisation techniques 7
    Deglycosylation for intact protein MS 8
    Irradiation setup 8
    Mass spectrometry data acquisition 8
    Preparative HPLC 9
    Preparation of Ab-PEG6-Ir conjugates 9
    Proteomic Data Analysis 9
3. Organic Synthesis 10
    DBCO-0-dFl 10
    NHboc-PEG6-dFl 12
    DBCO-PEG6-dFl 14
    Ir-PEG6-NHBoc 16
    Ir-PEG6-DBCO 20
    DBCO-bis-dFl 22
    Diazirine-PEG4-biotin 25

1. Supplementary Figures

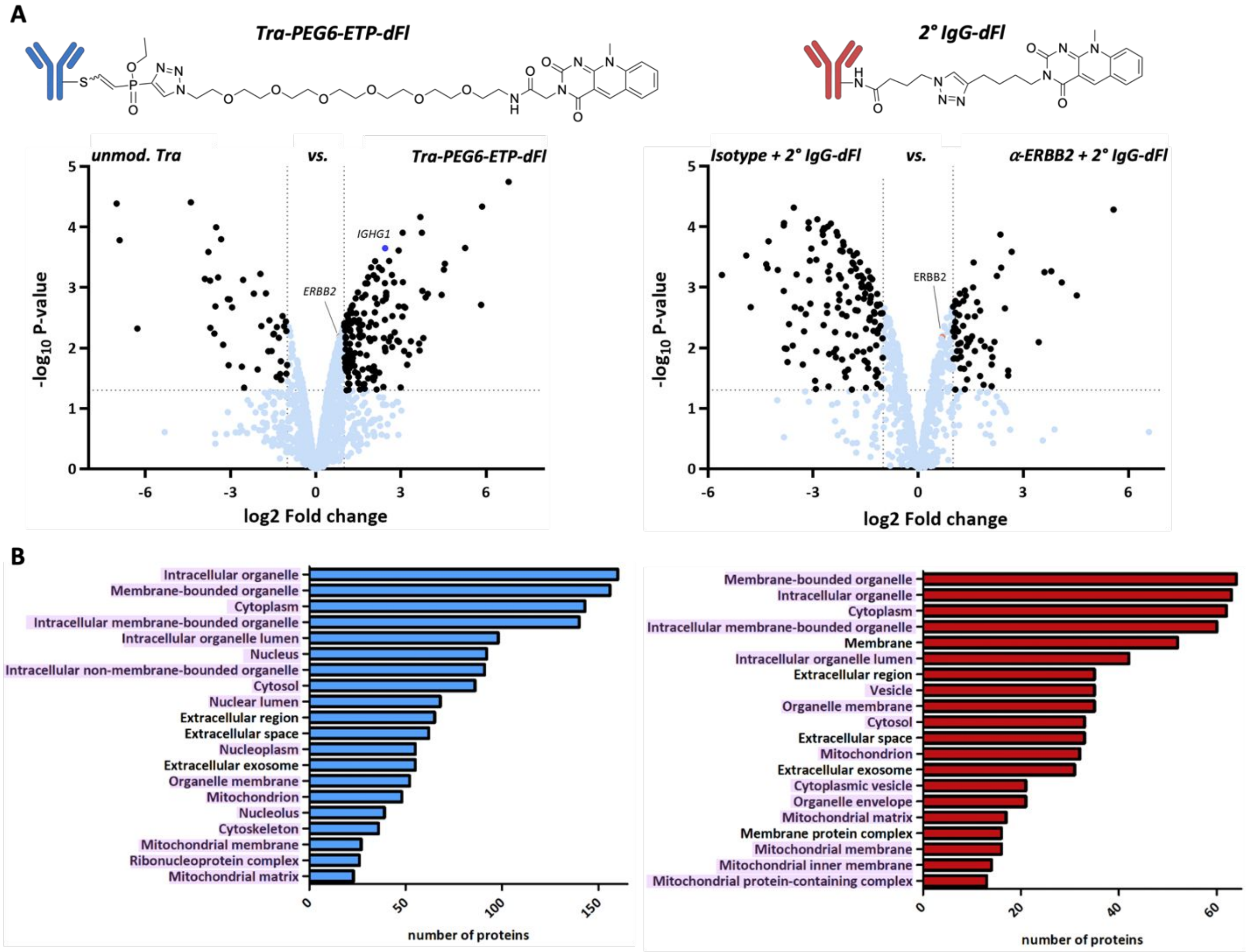

Figure S1: Proteomic data of PL experiments of treatments with the first set of antibody-dFl conjugates. (A) Structure of antibody-dFl conjugates Tra-PEG6-ETP-dFl and 2°IgG-dFl as well as volcano plots derived from respective treatments and proteomic measurements. Cell treatment took place as described in the experimental section. As indicated, treatment with unmodified Tra was applied as a negative control for Tra-PEG6-ETP-dFl. In case of 2°IgG-dFl, two consecutive antibody incubation steps took place: first with primary antibodies (Mouse HER2 antibody or Mouse IgG Isotype (negative control)), followed by polyclonal anti-mouse 2° IgG-dFl. HER2 (ERBB2, orange data point) was not significantly enriched in either data set. (B) Gene Ontology analysis of significantly enriched proteins relating to cellular compartment for Tra-PEG6-ETP-dFl (left, blue) and 2° IgG-dFl (right, red) treatment. In both data set, most proteins are attributed to intracellular cell compartments (highlighted in pink).

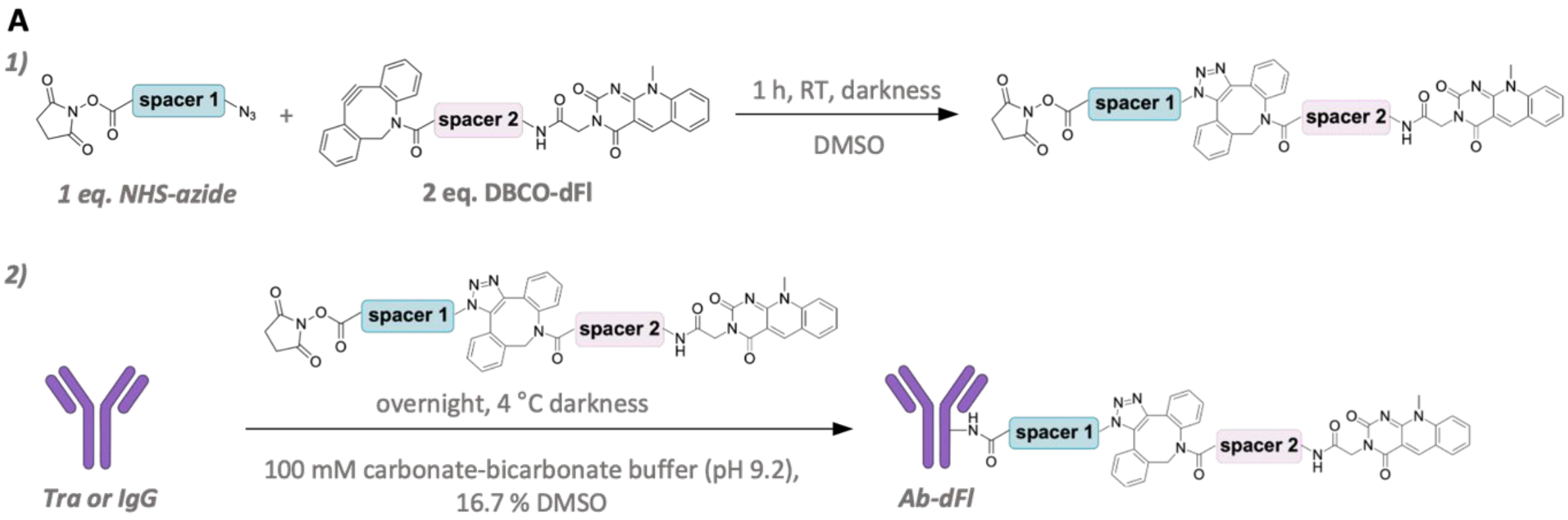

B

Antibody conjugate	NHS-azide	DBCO-dFl (for preparation see *3. Organic Synthesis*)
Ab-PEG0-dFl	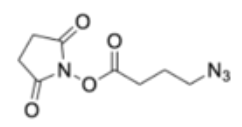 *azidobutyric acid NHS ester (CAS 943858-70-6)*	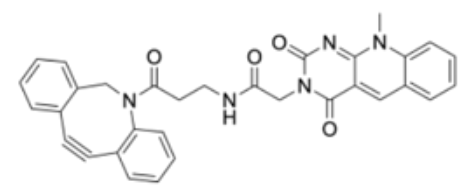 *DBCO-0-dFl*
Ab-PEG6-dFl	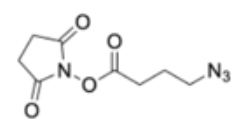 *azidobutyric acid NHS ester (CAS 943858-70-6)*	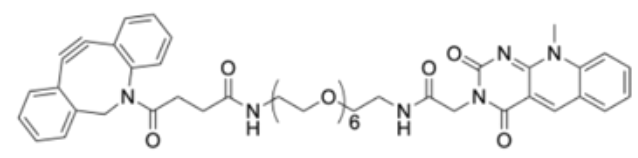 *DBCO-PEG6-dFl*
Ab-PEG12-dFl	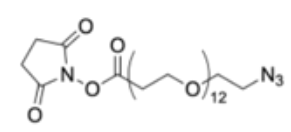 *azido-PEG12-NHS ester (CAS 2363756-50-5)*	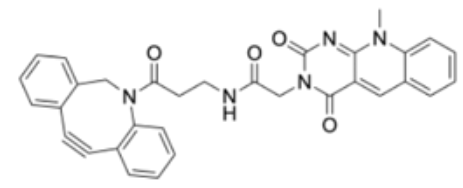 *DBCO-0-dFl*
Ab-bis-dFl	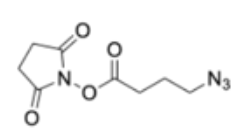 *azidobutyric acid NHS ester (CAS 943858-70-6)*	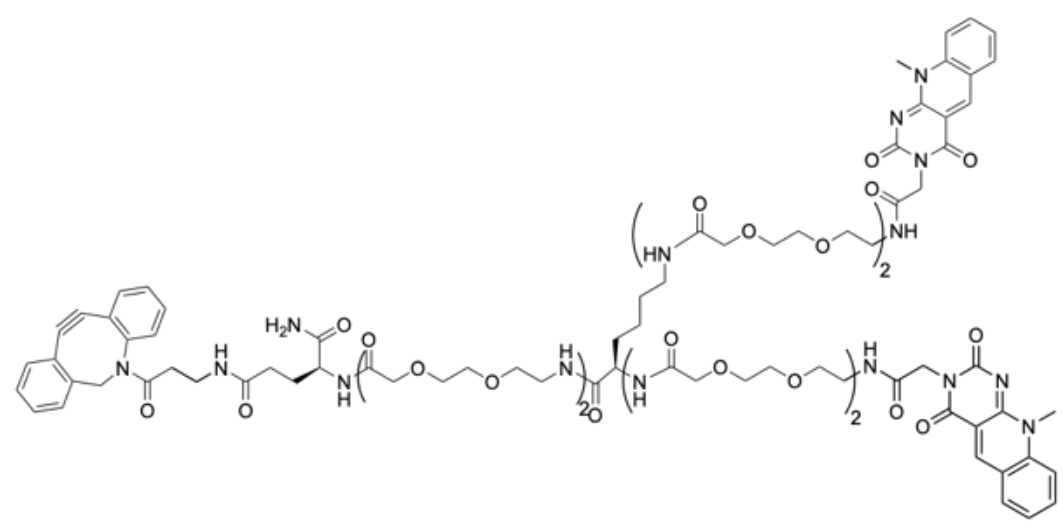 *DBCO-bis-dFl*

Figure S2: Antibody-dFl conjugate preparation. (A) Schematic reaction of the two-step conjugation protocol. NHS-azide and DBCO-dFl compounds are pre-incubated together before they are added to a buffered antibody (Tra or IgG) solution for the final conjugation. The full protocol can be found in the *Experimental Section* of the main text. For annotation of spacer 1 and 2 segments, see Figure 2.(B) Table indicating which compounds are used to achieve the respective Ab-dFl conjugate. NHS-azide have been purchased, while DBCO-dFl compounds were prepared according to the synthetic procedures elaborated under *3. Organic Synthesis* in this document.

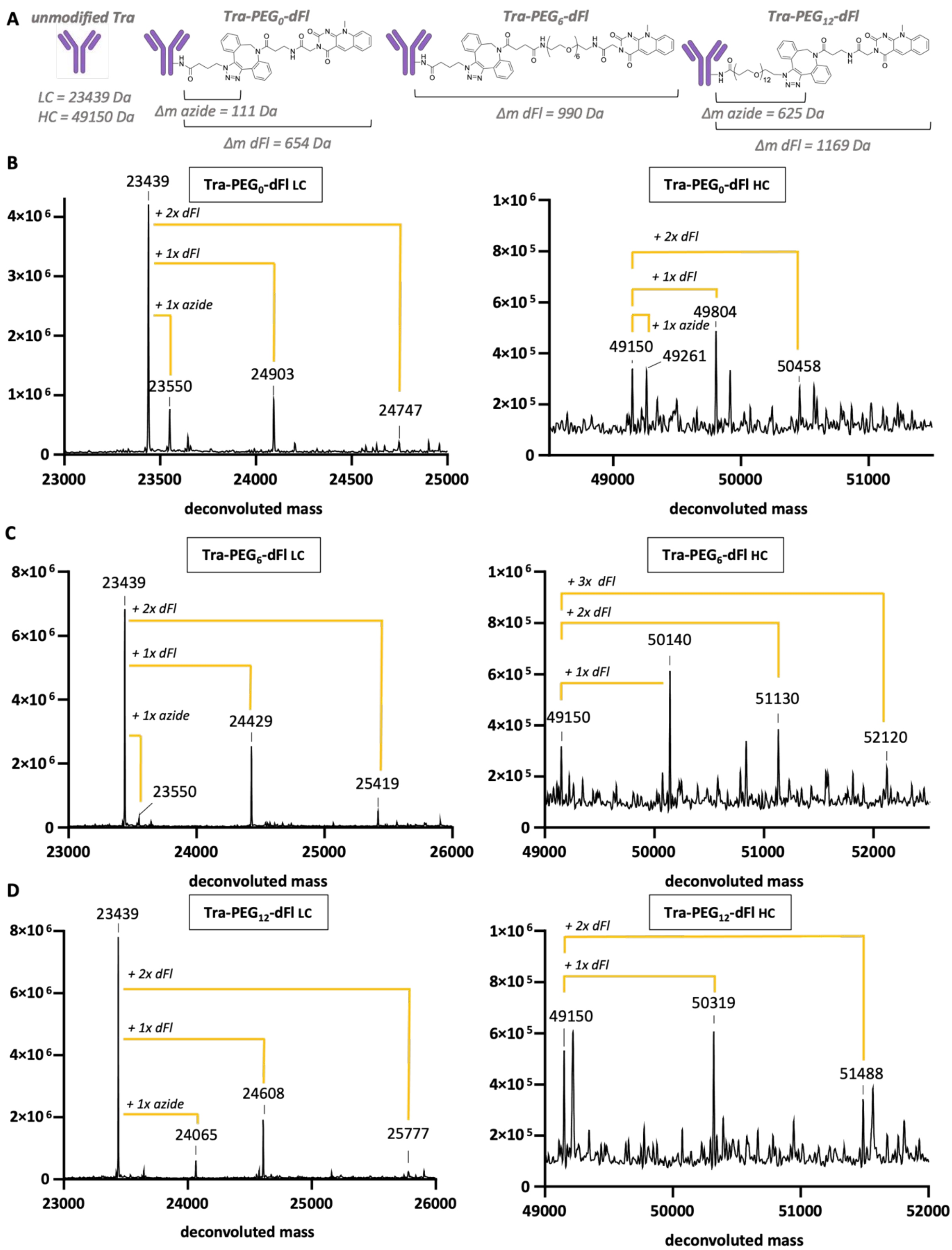

Figure S3: Evaluation of catalyst distribution with intact protein MS over the antibody's heavy chain (HC) and light chain (LC) of unbranched Tra-dFl conjugates. (A) Depiction of structures and mass additions of the respective Tra-dFl conjugates as well as unmodified Tra. (B-D) Deconvoluted intact protein mass spectra of reduced and deglycosylated Tra-dFl conjugates, in the respective mass ranges of modified LC and HC of the antibody. For the procedure and

measurement parameters see *2.2. Methods, Intact Protein MS*. Spectra were plotted using Graph Pad Prism 10 and peaks assigned to unmodified light or heavy chain as well as respective mass shifts of attached dFl-linker conjugates are labelled accordingly.

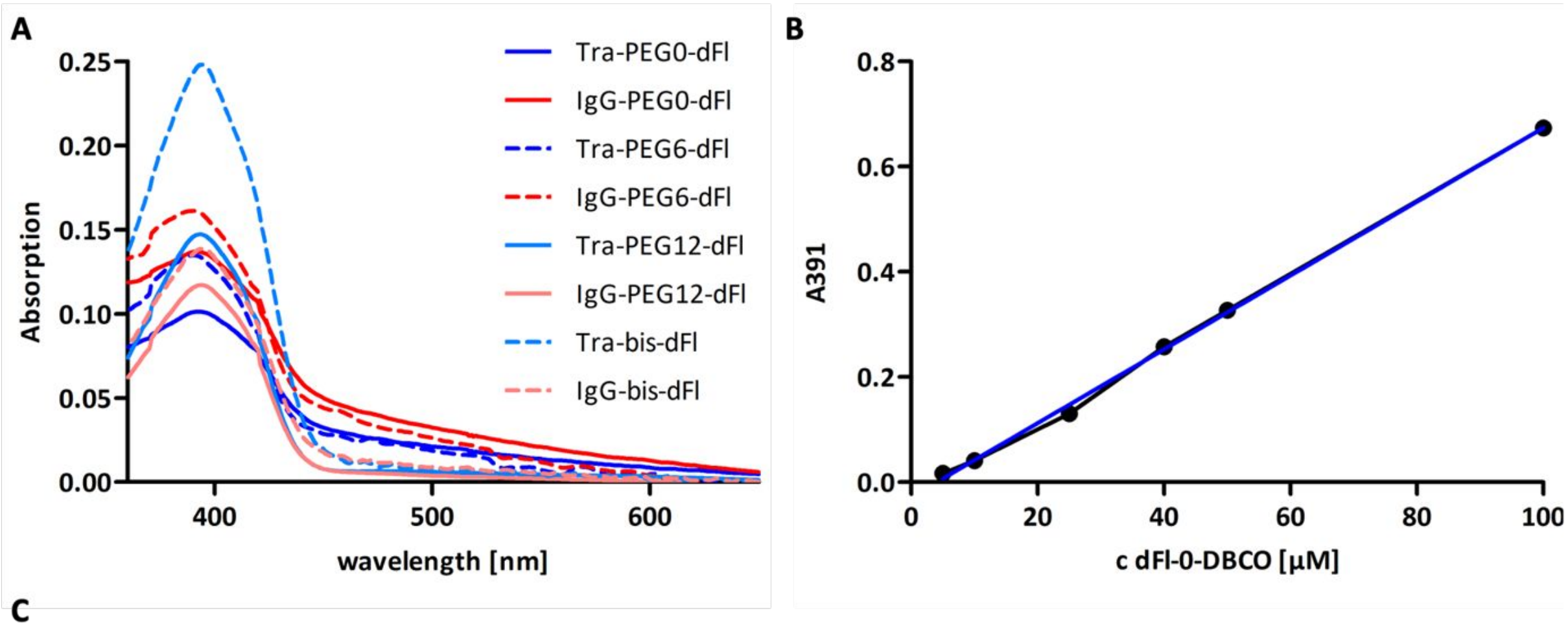

C

Ab-dFl conjugate	$c_{antibody}$ [µmol]	c_{dFl} [µmol]	dFl to antibody ratio
Tra-PEG0-dFl	2.25	17.54	7.8
IgG-PEG0-dFl	2.37	22.72	9.6
Tra-PEG6-dFl	3.35	22.42	6.7
IgG-PEG6-dFl	3.20	26.27	8.2
Tra-PEG12-dFl	3.13	24.13	7.7
IgG-PEG12-dFl	1.93	19.73	10.2
Tra-bis-dFl	4.14	38.54	9.3
IgG-bis-dFl	1.97	22.68	11.5

Figure S4: Characterisation data of antibody-dFl conjugates. (A) UV/vis spectra of all antibody-dFl conjugates in the range of 360-650 nm. (B) Standard curve of A391 (absorption measured at 391 nm) of free DBCO-0-dFl in the range of 5-100 µM in PBS containing 1% of DMSO. (C) Table containing the concentration of the antibody (obtained from bicinchoninic acid assay against a bovine serum albumin (BSA) standard dilution), dFl concentration interpolated from measured A391 of the conjugate and the standard curve in (B) as well as the respective ratio between dFl and antibody concentrations.

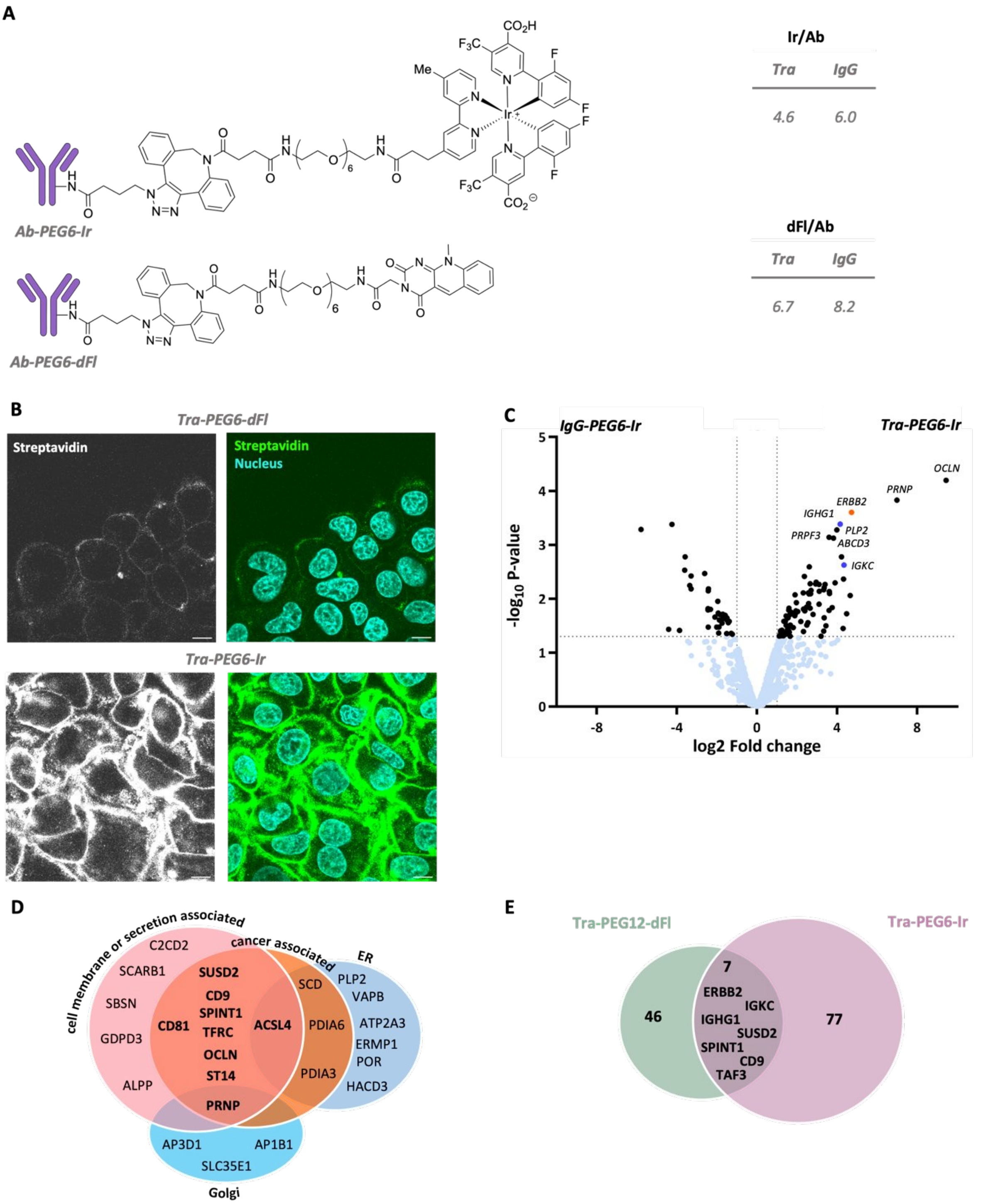

Figure S5: Benchmarking cell surface DarT labelling to Tra-Ir conjugate with coherent linker design. **A** Structure of the prepared and applied Ab-PEG6-Ir conjugates and depiction of Ab-PEG6-dFl for comparison. **B** Confocal fluorescence microscopy images revealing biotinylation through Streptavidin-Alexa Fluor™ 488 (white and green), and nuclei (cyan). Scale bar: 10 µm. **C** Volcano plot from streptavidin-enriched protein samples derived from cells treated with Tra-PEG6-Ir against the non-binding IgG-PEG6-Ir control. Significantly enriched proteins ($P < 0.05$; $\log_2$(Fold change) > 1) are coloured the following way: • orange ERBB2 (target) • blue IgG fragments (targeting moiety). The five proteins with highest log2 fold change are labelled. **D** Venn diagram displaying overlap between significantly enriched proteins derived from Tra-

PEG6-Ir treatment with reported connections to cancer mechanisms and their respective cellular localization. **E** Venn diagram of significantly enriched proteins from Tra-PEG12-dFl and Tra-PEG6-Ir treatment.

A

	log2 Fold change	-log_{10} (P-value
IGKC	4.69	3.84
FBXO10	4.64	6.23
IGHG1	4.08	4.70
TM9SF4	3.68	4.21
CCDC137	3.3	2.78
LBR	2.9	1.85
RPF1	2.86	4.13
LCN1	2.85	1.44
MRPL32	2.8	1.66
MRPL2	2.66	1.37
TACSTD2	2.65	1.79
ERBB2	2.61	5.29
OSTC	2.56	1.66
MBD1	2.55	2.35
ATP5MG	2.43	1.39
BCAM	2.37	1.83
RPS19BP1	2.36	1.51
H3-3A	2.3	2.07
PPIF	2.12	3.42
KRT27	2	1.53
RBM15	1.99	1.44
DHX8	1.95	2.22
ESYT2	1.95	1.49
SQOR	1.95	1.47
MGST3	1.93	1.51
TM9SF3	1.9	2.89
UBAP2L	1.88	1.43
SLC5A6	1.84	1.73
SLC30A1	1.84	1.58
NIPSNAP2	1.79	1.61
H2AC21	1.71	1.63
ARL6IP5	1.69	1.61
MAP7	1.65	1.73
ASPH	1.62	1.30
MRPS11	1.59	1.39
NDUFV2	1.51	1.70
RANBP2	1.46	2.13
NUCB1	1.34	1.38
TAF3	1.31	2.67
NOC4L	1.29	1.64
MT-CO2	1.25	1.43
CD9	1.22	2.05
TEX10	1.2	1.39
SPINT1	1.17	3.40
UTP14A	1.13	1.62
SUSD2	1.1	2.94
MPHOSPH10	1.08	2.03
CDC5L	1.05	1.84
PRPSAP2	1.04	1.91
MRPS5	1.04	1.68
MRPL54	1.02	2.10
RRP8	1.02	1.80
POP1	1.01	1.57

B

	log2 Fold change	-log_{10} (P-value
IGHG1	4.34	5.45
SNAP25	3.26	2.29
IGKC	3.09	3.95
ERBB2	2.41	5.28
SART1	2.21	1.59
FLNA	2.05	1.63
SUB1	1.98	2.24
FLNC	1.9	1.77
PRPF6	1.61	2.46
CALR	1.3	2.46
HP1BP3	1.19	2.01

C

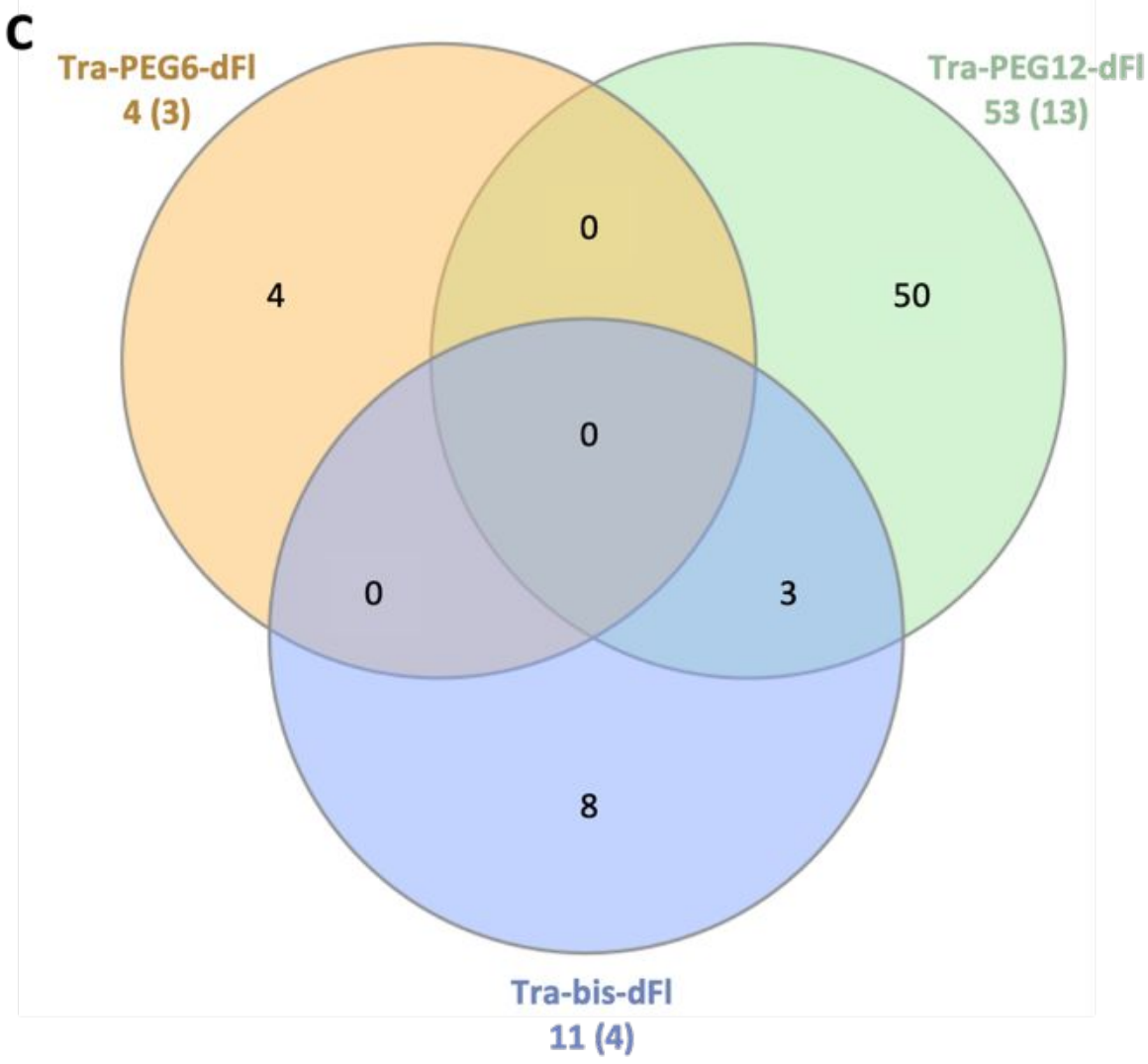

Figure S6: Significantly enriched proteins from HER2 targeted PPL on SK-BR-3 cells using (A) Tra-PEG12-dFl or (B) Tra-bis-dFl and a Venn diagram comparing all significantly enriched proteins for the Tra-dFl constructs with the number of cell surface annotated hits in brackets (C).Proteins were obtained after treatment of cells according to the procedures described in the Experimental Section and section 2.2. ‘Proteomic Data Analysis’. All mass spectrometry proteomics data have additionally been deposited by the PRIDE partner repository and are available via the ProteomeXchange Consortium with the dataset identifier PXD076830.

2. Materials and Methods

2.1 Reagents and Materials

All reagents for organic synthesis were purchased from either Fisher Scientific (DE), BLD Pharma (DE), Sigma-Aldrich (DE) or TCI Chemicals (BE) in the highest purity available and used without further purification. Solvents were purchased from Avantor (DE) or Fisher Scientific (DE) in Laboratory grade, HPLC and LC/MS grade, respectively.

Other materials and their respective vendors are listed below:

Material	Vendor
**Antibodies**	
Goat anti-Mouse IgG (AP124)	Sigma-Aldrich (DE)
HER-2 Monoclonal Antibody (e2-4001)	Invitrogen
Mouse IgG1 kappa Isotype Control (P3.6.2.8.1)	Invitrogen
Trastuzumab (Herceptin 1289787)	Medizone Germany
**Enzymes**	
PNGase F	New England Biolabs
Trypsin	Promega
**Other**	
DMEM (Dulbecco's Modified Eagle Medium) F12	Biowest
Hydrophobic and hydrophilic Carboxylate-Modified Magnetic Beads (Sera-Mag™)	Cytiva
Protease Inhibitor Cocktail (cOmplete™, EDTA-free)	Sigma-Aldrich (DE)
Streptavidin Magnetic Beads	New England Biolabs

2.2 Methods

Cell culture

Cells were maintained at 37 °C, 5 % CO_2 in a humidified atmosphere and split every two days or until confluency reached 90 %. SK-BR-3 (LMU München Biozentrum) cells were maintained in Dulbecco's Modified Eagle Medium (DMEM) medium with F-12 modification, stable L-glutamine, and 10 % fetal calf serum (FCS).

Characterisation techniques

NMR spectra were measured with a Bruker UltrashieldTM 600 MHz (Bruker Corp., USA) at room temperature. The resonance multiplicity is abbreviated as: s (singlet), d (doublet), t (triplet), q (quadruplet), quint (quintet), sext (sextet), sep (septet), m (multiplet) and br (broad). The chemical shift δ is expressed in ppm and the coupling constant J is in Hz. The chemical shifts are referenced to the residual solvent peak as the internal standard. High

resolution mass spectra (HRMS) were recorded on an Acquity UPLC® system with a Xevo® G2-XS Quadrupole time-of-flight (QTof) mass spectrometer (both Water Corp., USA). For small molecules and peptides, an ACQUITY UPLC BEH C18 column (1.7 µm, 2.1 mm x 50 mm) was used with the following gradient: A = H_2O + 0.01% formic acid, B = MeCN + 0.01% formic acid, 10-90% B 0-5.5 min. For intact protein MS an ACQUITY UPLC Protein BEH C4 Column, (300 Å, 1.7 µm, 2.1 mm x 50 mm) was used. Proteins were eluted with a flow rate of 0.3 mL/min. The following gradient was used: A: 0.01% formic acid in H_2O; B: 0.01% formic acid in MeCN. 5-95% B 0-5.5 min. Mass analysis was conducted with a Waters XEVO G2-XS QTof analyzer. Raw data was analyzed with MaxEnt 1. UV-Vis absorption spectra were obtained with a JASCO Spectrophotometer.

Deglycosylation for intact protein MS

Tra-dFl conjugates were reduced and deglycosylated prior to intact protein MS using a solution containing dithiothreitol (DTT) and PNGase F. In general, 1 µL of a 5 mg/mL Tra-dFl solution was diluted with 180 µL PBS and 20 µL of a DTT solution in PBS (100 mM) as well as 1 µL of PNGase F. Reduction and deglycosylation of the antibody took place over 2-3 h at 37 °C.

Irradiation setup

The light source used for irradiating cells growing on glass-bottom IBIDI slides or in 6 cm cell culture dishes was an EvoluChem 450 nm Blue LED (30W, λ_{max} = 445 nm, Hepatochem, US). Irradiation took place in a cold room (4 °C) using one LED lamp per slide or dish at approximately 5 cm above the dish.

Mass spectrometry data acquisition

LC–MS/MS analysis was performed using either an UltiMate 3000 system coupled online to an Orbitrap Fusion mass spectrometer or a Vanquish Neo system coupled to an Orbitrap Exploris 480 mass spectrometer (Thermo Fisher Scientific). For both setups, samples were loaded onto a PepMap C18 trap column (0.075 mm × 50 mm, 3 µm particle size, 100 Å pore size; Thermo Fisher Scientific) and separated on a 50 cm analytical column (in-house packed with Poroshell 120 EC-C18, 2.7 µm; Agilent Technologies) at a flow rate of 250 nL/min. For Orbitrap Fusion measurements, mobile phase A consisted of 0.1 % formic acid in water and mobile phase B of 0.1 % formic acid in MeCN. Peptides were separated using a 104 min gradient: 4–5 % B (0–8 min), 5–25 % B (8–74 min), 25–28 % B (74–80 min), 28–31 % B (80–86 min), 31–36 % B (86–92 min), 36–40 % B (92–95 min), 40–50 % B (95–96 min), 50–80 % B (96–101 min), held at 80 % B (101–104 min), and re-equilibrated to 4 % B. MS1 survey scans were acquired in the Orbitrap (m/z 375–1500) at 120,000 resolution (AGC target 4×10^5, maximum injection time 50 ms). Precursors with charge states 2–4 were isolated using a 1.6 m/z window, with dynamic exclusion set to 40 s (± 10 ppm), and fragmented by HCD (NCE 30 %). MS2 spectra were acquired in the linear ion trap (rapid mode; AGC target 1×10^4, maximum injection time 35 ms) with a cycle time of 1 s. For Orbitrap Exploris 480 measurements, mobile phase A contained 1 % MeCN and 0.1 % formic acid in water, and mobile phase B contained 80 % MeCN with 0.1% formic acid. Peptides were separated over a

118 min gradient: 4–30 % B (0–85 min), 30–50 % B (85–105 min), and 50–99 % B (105–118 min). MS1 scans were acquired in the Orbitrap at 120,000 resolution over an m/z range of 375–1200 (AGC target 3×10^6, automatic maximum injection time). Precursors with charge states 2–4 were isolated with a 1.6 m/z window and subjected to HCD fragmentation (NCE 30 %) with a dynamic exclusion of 20 s (± 10 ppm). MS2 spectra were acquired in the Orbitrap at 15,000 resolution (AGC target 5×10^4, automatic maximum injection time) in 2 s cycles.

Preparative HPLC

Preparative HPLC was performed on a Shimadzu prominence HPLC system (Shimadzu Corp., Japan) containing a CBM20A communication bus module, a FRC-10A fraction collector, two pumps LC-20AP, and a SPD-20A UV/VIS detector, using a VP250/21 Macherey-Nagel Nucleodur C18 HTec Spum column (Macherey-Nagel GmbH & Co. Kg, Germany). The following gradient was used: (A = H_2O + 0.1 % TFA, B = MeCN + 0.1 % TFA), flow rate 10 mL/min, 5 % B 0-5 min, 5-80 % B 5-60 min, 80-95 % B 60-70 min.

Preparation of Ab-PEG6-Ir conjugates

Ab-PEG6-Ir conjugates were prepared analogously to Ab-dFl conjugates (see *Experimental Section* in the article). Azidobutyric acid NHS ester and Ir-PEG6-DBCO (for synthesis see below) were used as reagents in this case. The degree of modification was determined by dividing the micromolar concentration of Ir (determined by absorbance measurement at 350 nm and comparison to a standard dilution of free Ir-PEG6-DBCO) by the micromolar concentration of the antibody (determined through a bicinchoninic acid assay against a bovine serum albumin (BSA) standard dilution).

Proteomic Data Analysis

Raw data from LC-MS/MS measurements were analysed with FragPipe (v22.0) using the built-in LFQ-MBR workflow and the whole human proteome as the search space. The following MSFragger[1] settings were applied: Precursor mass tolerance: ±20 ppm; Fragment mass tolerance: ±20 ppm; Mass calibration and parameter optimization enabled; Isotope error: 0/1/2; Enzyme: stricttrypsin (cuts after K and R), Peptide length: 7–50 AA; Peptide mass range: 500–5000 Da; Variable modifications: Oxidation (M, 15.9949 Da, up to 3×), Acetylation (N terminal, 42.0106 Da, 1x); Carbamidomethylation of C was set as fixed modification (57.02146 Da). Validation was performed using Percolator[2] and ProteinProphet[3], and an FDR of 1 % was applied. For MS1 Quantification, IonQuant[4] was used for LFQ (including MaxLFQ), with MBR (FDR of 1 %) and normalisation across the runs enabled. The resulting files were further processed using FragPipe-Analyst[5] as follows: Proteins must be quantified in >66% of the files for at least one condition and in >66% of the files globally. Median-centred normalization of the MaxLFQ intensity extracted from FragPipe results was enabled, as well as Perseus-type imputation and Benjamini–Hochberg-type FDR correction. The following significance cut-offs were used: $\log_2$(fold change) > 1; $P < 0.05$. GO cellular component enrichment analysis was carried out using the default settings in ShinyGO 0.80[6], importing only significantly enriched proteins from either of the two conditions against a complete human background. Volcano plots were created using GraphPad Prism 10 (10.0.0).

3. Organic Synthesis

For the synthesis of all dFl-DBCO compounds used to prepare antibody-dFl conjugates (DBCO-0-dFl, DBCO-PEG6-dFl and DBCO-bis-dFl), dFl-COOH (2-(10-Methyl-2,4-dioxo-4,10-dihydropyrimido[4,5-b]quinolin-3(2H)-yl)acetic acid) was used as the starting compound, the preparation of which is described elsewhere.[7] No unexpected or unusually high safety hazards were encountered.

DBCO-0-dFl

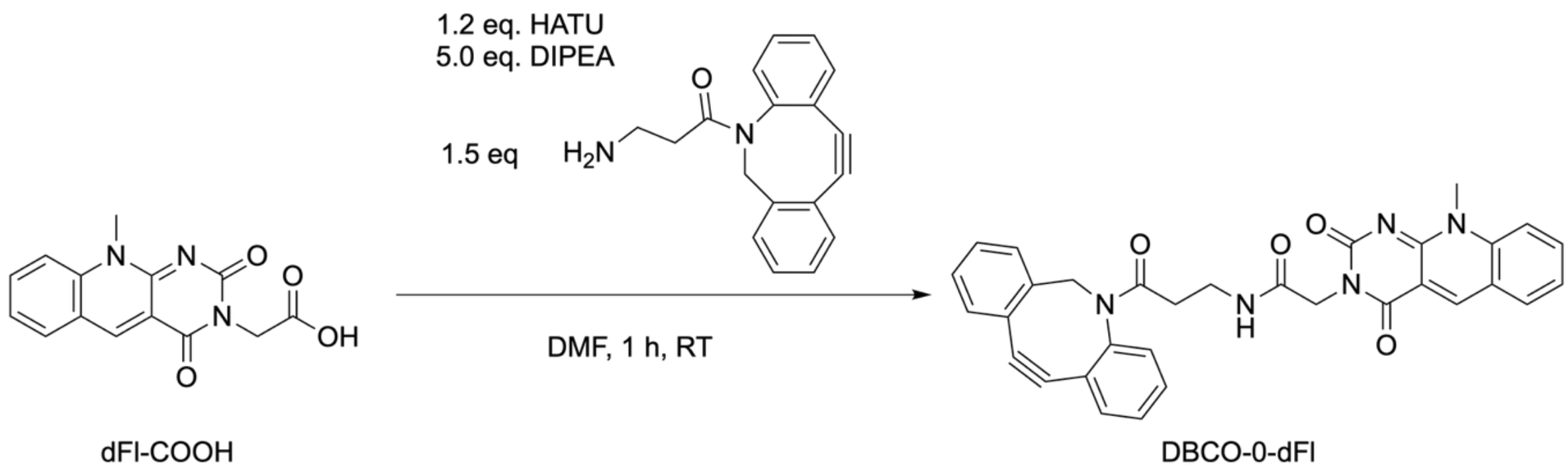

For the synthesis of DBCO-0-dF, 15 mg (52.6 µmol) of dFl-COOH was dissolved in 150 µL DMF. 24 mg Hexafluorophosphate Azabenzotriazole Tetramethyl Uronium (HATU; 1.2 eq., 63.1 mg) were dissolved in 150 µL DMF in a different reaction vessel, and 44.7 µL *N,N*-diisopropylethylamine (DIPEA; 5 eq., 34 mg) were added. After mixing, this solution was combined with the dFl-COOH solution. To start the reaction, a solution of 21.8 mg dibenzocyclooctyne-amine (1.5 eq., 78.9 µmol) in 150 µL DMF was added to the activated acid. The reaction mixture stirred at room temperature for one hour and was subsequently purified by preparative HPLC. After lyophilisation, the product was collected as a yellow solid (12.84 mg, 23.2 µmol, 45 %).

^{1}H NMR (600 MHz, DMSO) δ = 9.05 (s, 1H), 8.24 – 8.23 (m, 1H), 7.98 (q, J = 6.3 Hz, 3H), 7.64 – 7.61 (m, 2H), 7.58 (ddd, J = 8.0, 6.1, 1.8 Hz, 1H), 7.51 (ddd, J = 8.6, 4.1, 1.6 Hz, 2H), 7.48 – 7.45 (m, 1H), 7.35 (dddd, J = 29.2, 16.1, 6.6, 5.0 Hz, 3H), 5.05 (dd, J = 14.2, 8.5 Hz, 1H), 4.44 – 4.33 (m, 2H), 4.08 (s, 2H), 3.76 (s, 1H), 3.20 – 3.13 (m, 1H), 2.97 (ddt, J = 13.8, 8.6, 5.4 Hz, 1H) ppm.

^{13}C NMR (151 MHz, DMSO) δ = 170.60, 167.29, 161.78, 158.79, 156.47, 155.79, 151.87, 148.78, 142.83, 141.18, 136.05, 134.17, 132.87, 132.19, 130.11, 129.60, 128.72, 128.52, 128.21, 127.28, 125.70, 125.01, 122.96, 121.87, 121.52, 117.29, 114.99, 114.75, 108.57, 55.34, 43.44, 35.58, 34.48, 32.65 ppm.

HRMS (ESI) m/z: $[M+H]^+$ for $C_{32}H_{25}N_5O_4$ Calculated 544.1980; Observed 544.2000.

^{1}H NMR spectrum of DBCO-0-dFl:

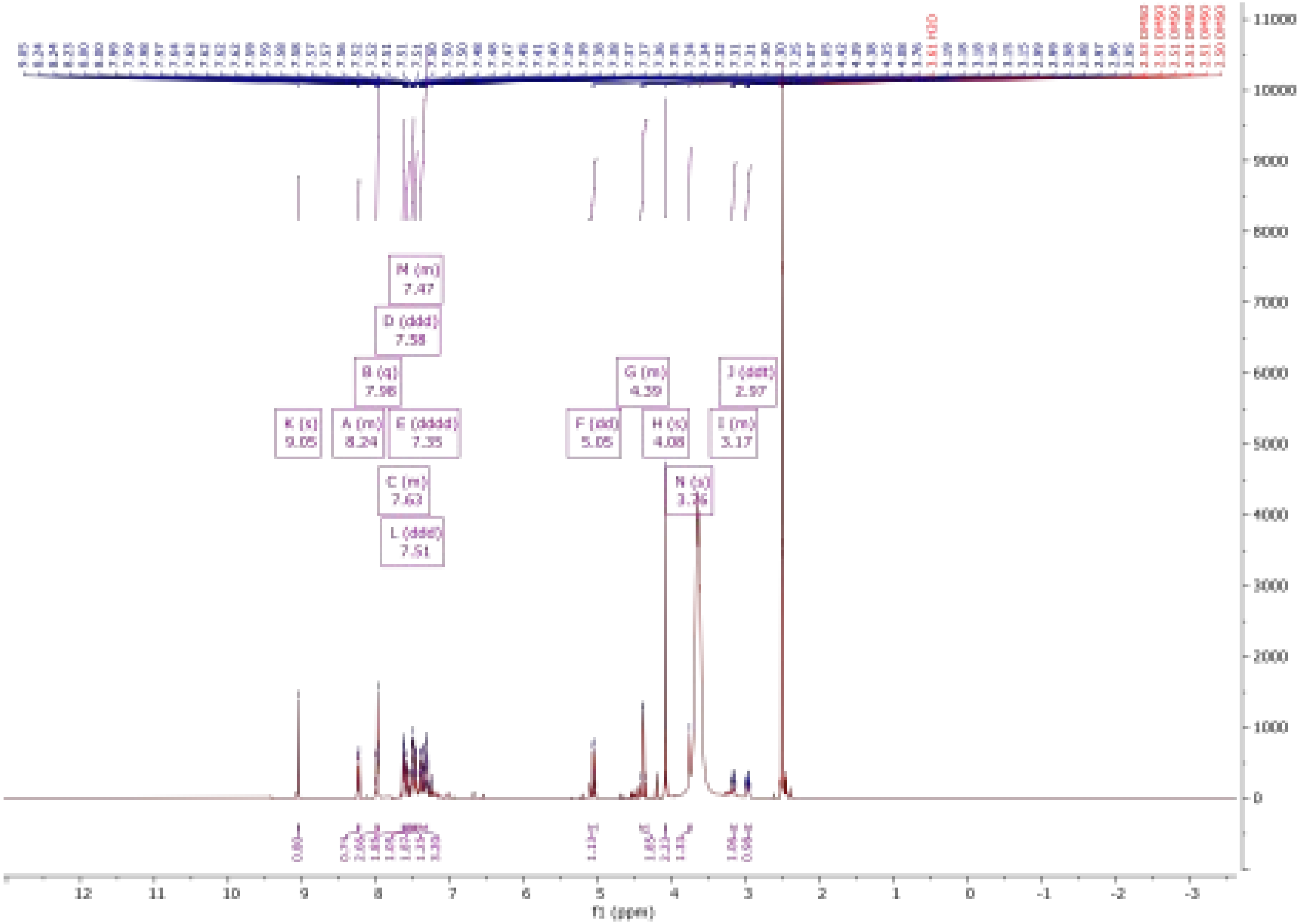

^{13}C NMR spectrum of DBCO-0-dFl:

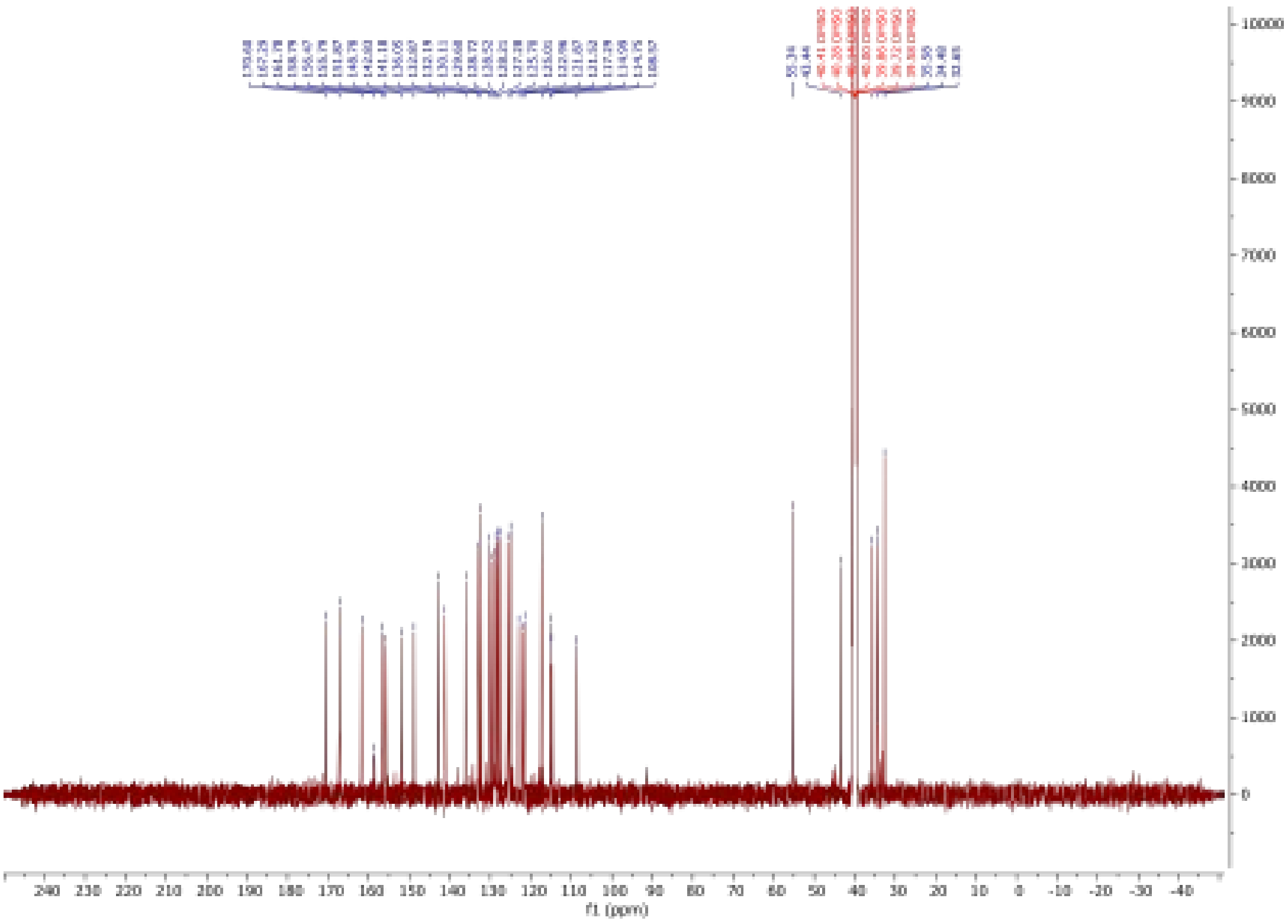

NHboc-PEG6-dFl

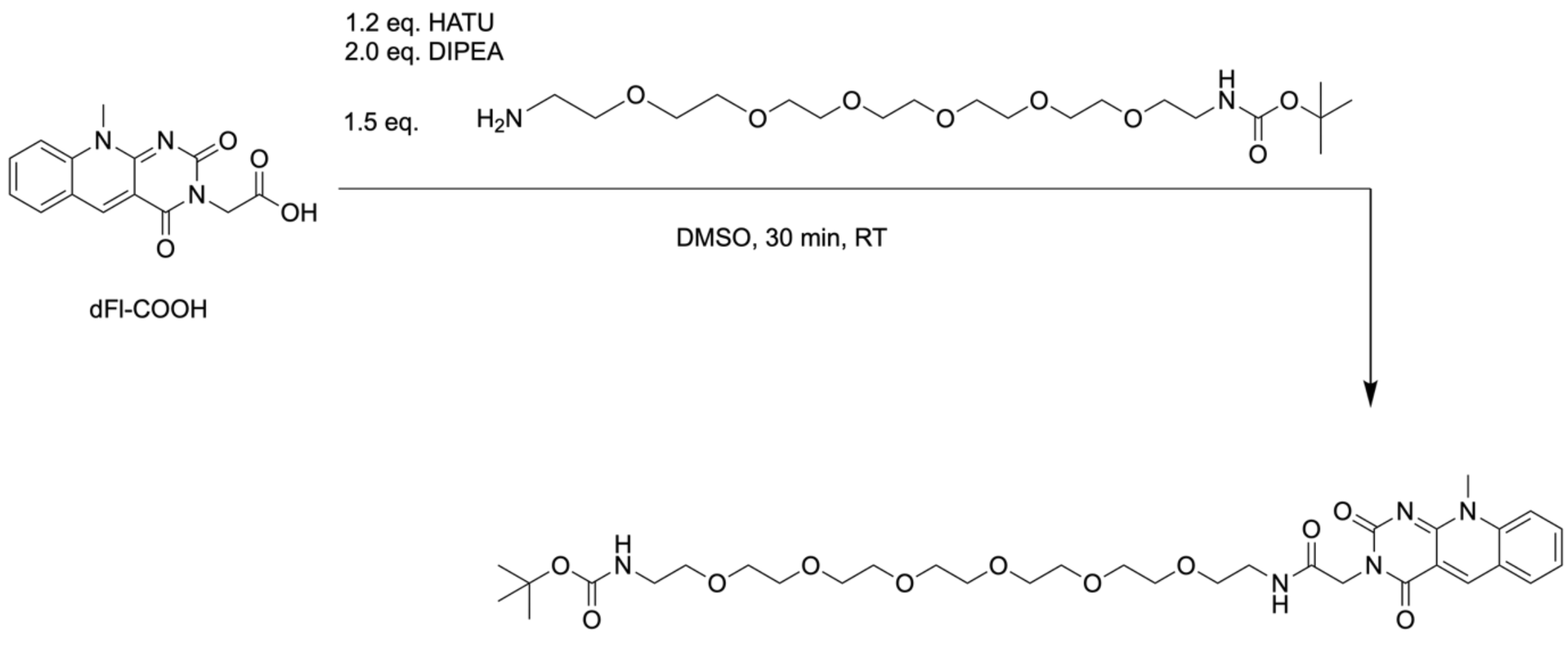

20 mg (70.1 µmol) dFl-COOH are dissolved in 250 µL DMSO and a mixture of 32 mg (1.2 eq., 84.12 µmol) HATU and 23.89 µL (2 eq., 140.2 µmol, 18.16 mg) DIPEA in 250 µL DMSO was added to the solution. Immediately, a solution of 44.6 mg NH_2-PEG6-NH-boc (1.5 eq., 105.2 µmol) dissolved in 500 µL DMSO was added and the reaction was stirred at room temperature for 30 min. The resulting product was then purified by preparative HPLC and dried through lyophilization. The resulting product is of gel-like consistency (37.83 mg, 54.7 µmol, 78 %).

^{1}H NMR (600 MHz, DMSO) = δ 9.07 (s, 1H), 8.27 – 8.22 (m, 1H), 8.12 (t, *J* = 5.7 Hz, 1H), 7.99 (d, *J* = 1.7 Hz, 1H), 7.58 (ddd, *J* = 7.9, 6.0, 2.1 Hz, 1H), 4.49 (s, 2H), 4.09 (s, 3H), 3.52 (dd, *J* = 8.5, 3.5 Hz, 20H), 3.42 (d, *J* = 5.9 Hz, 2H), 3.38 (s, 2H), 3.23 (q, *J* = 5.9 Hz, 2H), 3.06 (q, *J* = 6.0 Hz, 2H), 1.37 (s, 9H).

^{13}C NMR (151 MHz, DMSO) = δ 167.48, 161.82, 156.53, 155.85, 142.81, 141.21, 136.04, 132.19, 124.99, 121.52, 117.30, 115.03, 78.04, 70.26, 69.98, 69.63, 69.51, 43.48, 40.43, 40.30, 40.16, 40.02, 39.88, 39.74, 39.60, 39.22, 32.64, 28.71.

HR-MS (ESI$^+$) for $C_{33}H_{49}N_5O_{11}$ $[M+H]^+$ calculated: 692.3502 observed: 692.3510.

^{1}H NMR spectrum of NHboc-PEG6-dFl:

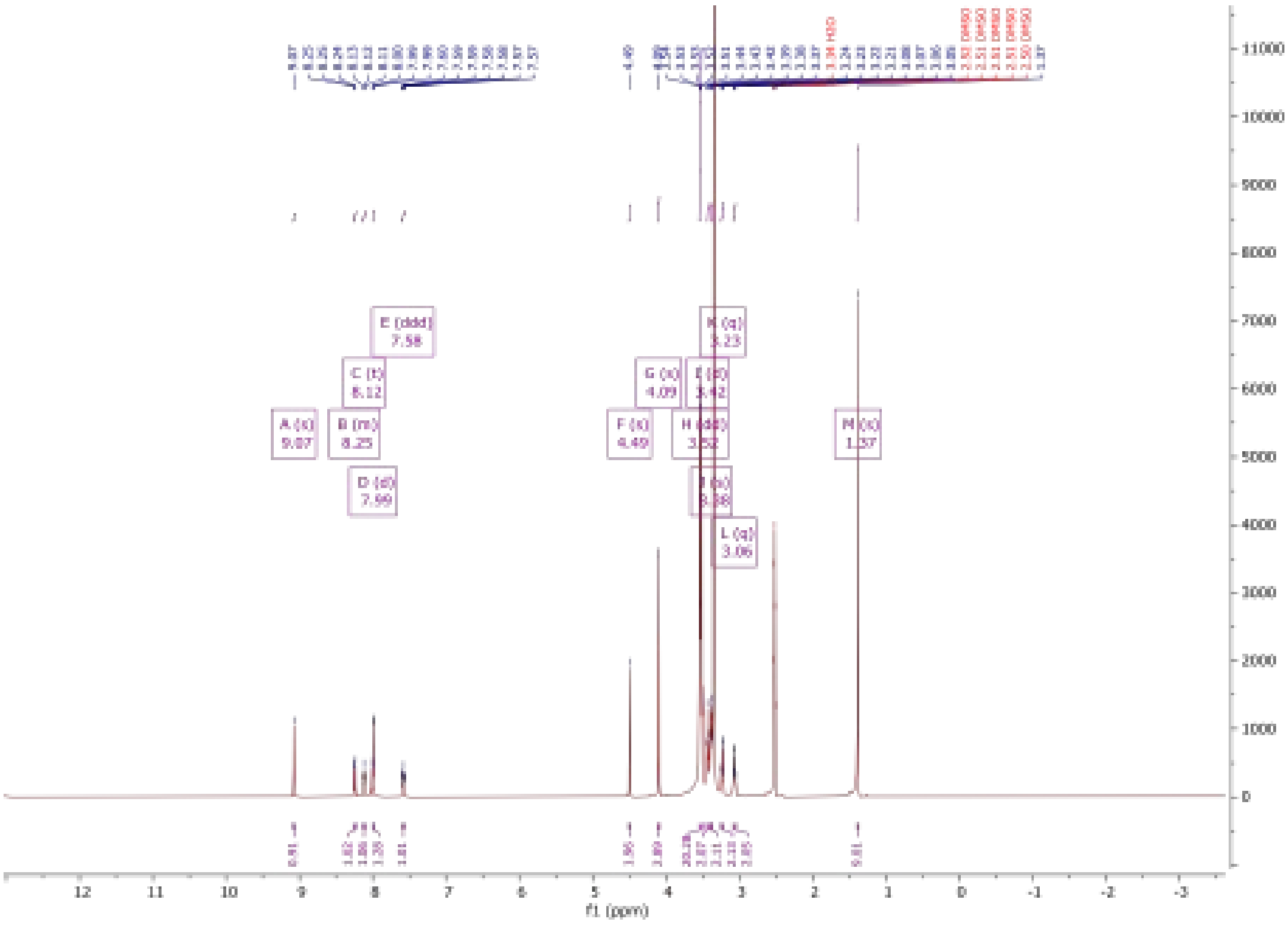

^{13}C NMR spectrum of NHboc-PEG6-dFl:

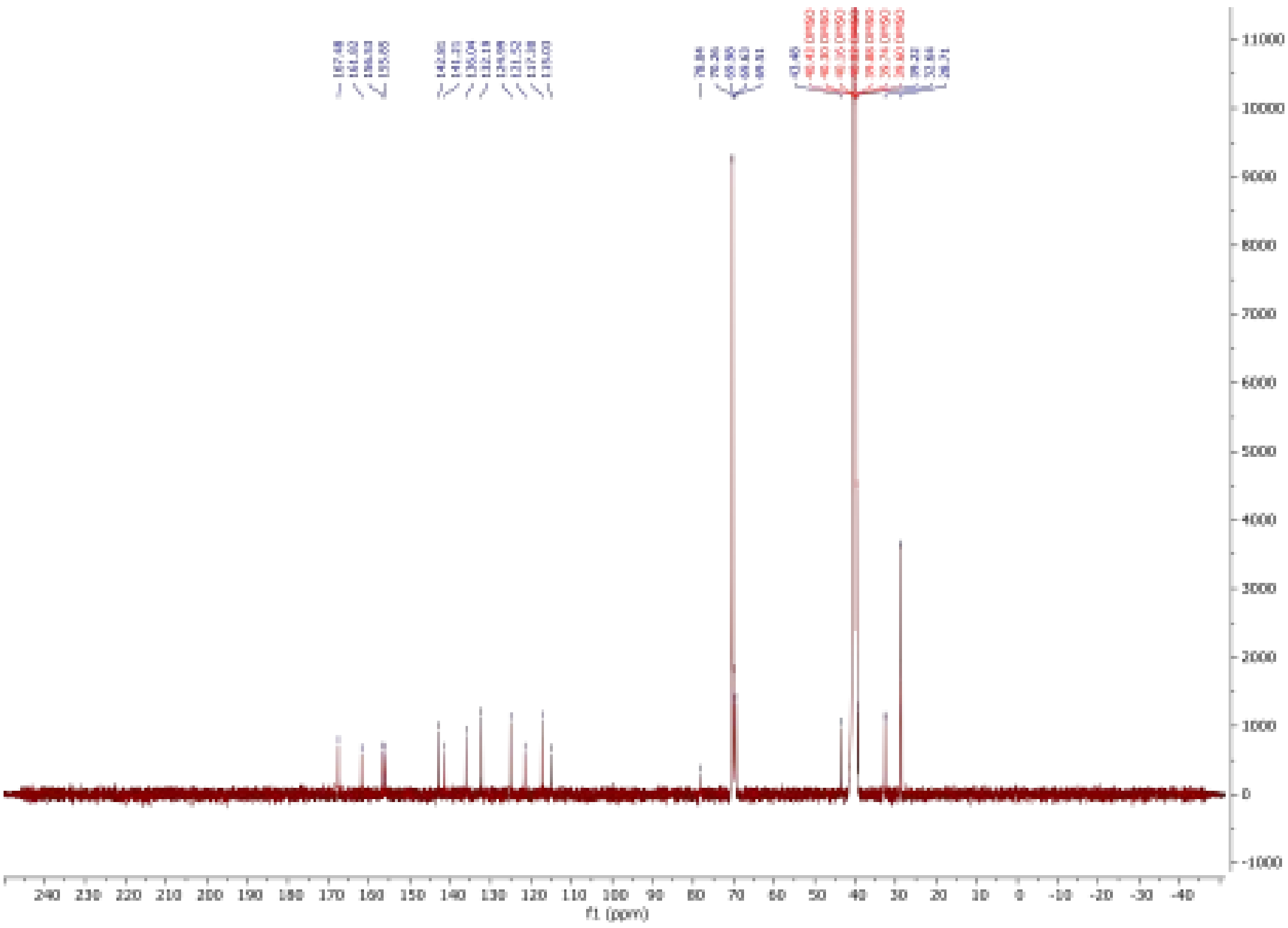

DBCO-PEG6-dFl

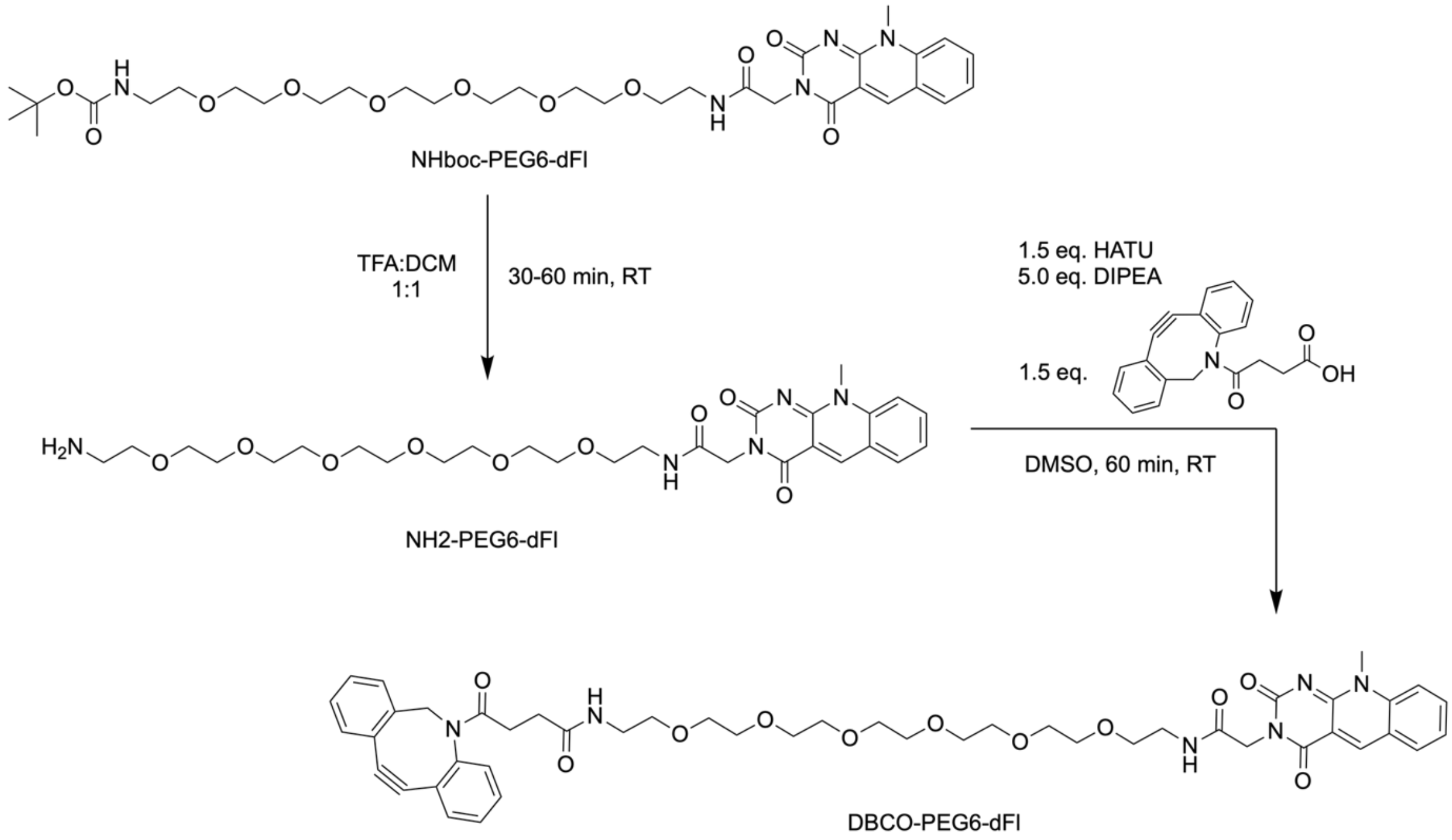

To deprotect NHboc-PEG6-dFl, the compound (30 mg, 43.4 µmol) was dissolved in 2 mL of 40 % trifluoroacetic acid (TFA) in DCM plus 1 % triisopropylsilan (TIS) and stirred for 30-60 min at room temperature. The solvents were evaporated under a N_2 stream and the deprotected amine precipitated using diethylether. A pellet was formed through centrifugation (10000 g, 10 min, 4 °C) and dried by decanting the ether and applying a N_2 stream again. The resulting compound was dissolved in 250 µL DMSO. To a mixture of 19.88 mg dibenzocyclooctyne-acid (1.5 eq., 65.1 µmol) and 24.75 mg HATU (1.5 eq., 65.1 µmol) dissolved in 250 µL DMSO were added 36.90 µL (5 eq., 217 µmol, 28.05 mg) of DIPEA. The resulting mixture was combined with the amine solution and stirred at room temperature for 60 min. The product was purified by preparative HPLC, lyophilized and obtained as a yellow solid (23.14 mg, 33.4 µmol, 77 %)

^{1}H NMR (600 MHz, DMSO) δ = 9.08 (s, 1H), 8.27 – 8.22 (m, 1H), 8.11 (t, *J* = 5.7 Hz, 1H), 8.00 (dd, *J* = 5.9, 1.5 Hz, 2H), 7.73 (t, *J* = 5.7 Hz, 1H), 7.68 (dd, *J* = 7.5, 1.6 Hz, 1H), 7.62 (dd, *J* = 7.5, 1.4 Hz, 1H), 7.58 (ddd, *J* = 8.0, 5.9, 2.2 Hz, 1H), 7.53 – 7.43 (m, 3H), 7.36 (dtd, *J* = 21.7, 7.4, 1.5 Hz, 2H), 7.29 (dd, *J* = 7.4, 1.6 Hz, 1H), 5.03 (d, *J* = 14.2 Hz, 1H), 4.49 (s, 2H), 4.09 (s, 3H), 3.61 (d, *J* = 13.9 Hz, 2H), 3.58 – 3.46 (m, 20H), 3.45 (dd, *J* = 5.6, 3.1 Hz, 2H), 3.42 (t, *J* = 5.9 Hz, 2H), 3.30 (td, *J* = 5.9, 2.2 Hz, 2H), 3.22 (q, *J* = 5.8 Hz, 2H), 3.11 – 3.05 (m, 2H).

^{13}C NMR (151 MHz, DMSO) δ = 171.55, 171.50, 167.47, 161.81, 155.84, 152.08, 148.90, 142.83, 141.21, 136.04, 132.88, 132.19, 130.08, 129.38, 128.58, 128.44, 128.13, 127.24, 125.61, 124.99, 123.00, 121.88, 121.53, 117.30, 115.04, 114.69, 108.62, 70.24, 70.19, 70.15, 70.12, 70.01, 69.51, 69.46, 55.36, 43.47, 40.56, 40.44, 40.30, 40.16, 40.02, 39.88, 39.74, 39.60, 39.21, 38.96, 32.65, 30.82, 30.18.

HR-MS (ESI$^+$) for $C_{47}H_{54}N_6O_{11}$ $[M+H]^+$ calculated: 879.3924 observed: 879.3858.

^{1}H NMR spectrum of DBCO-PEG6-dFl:

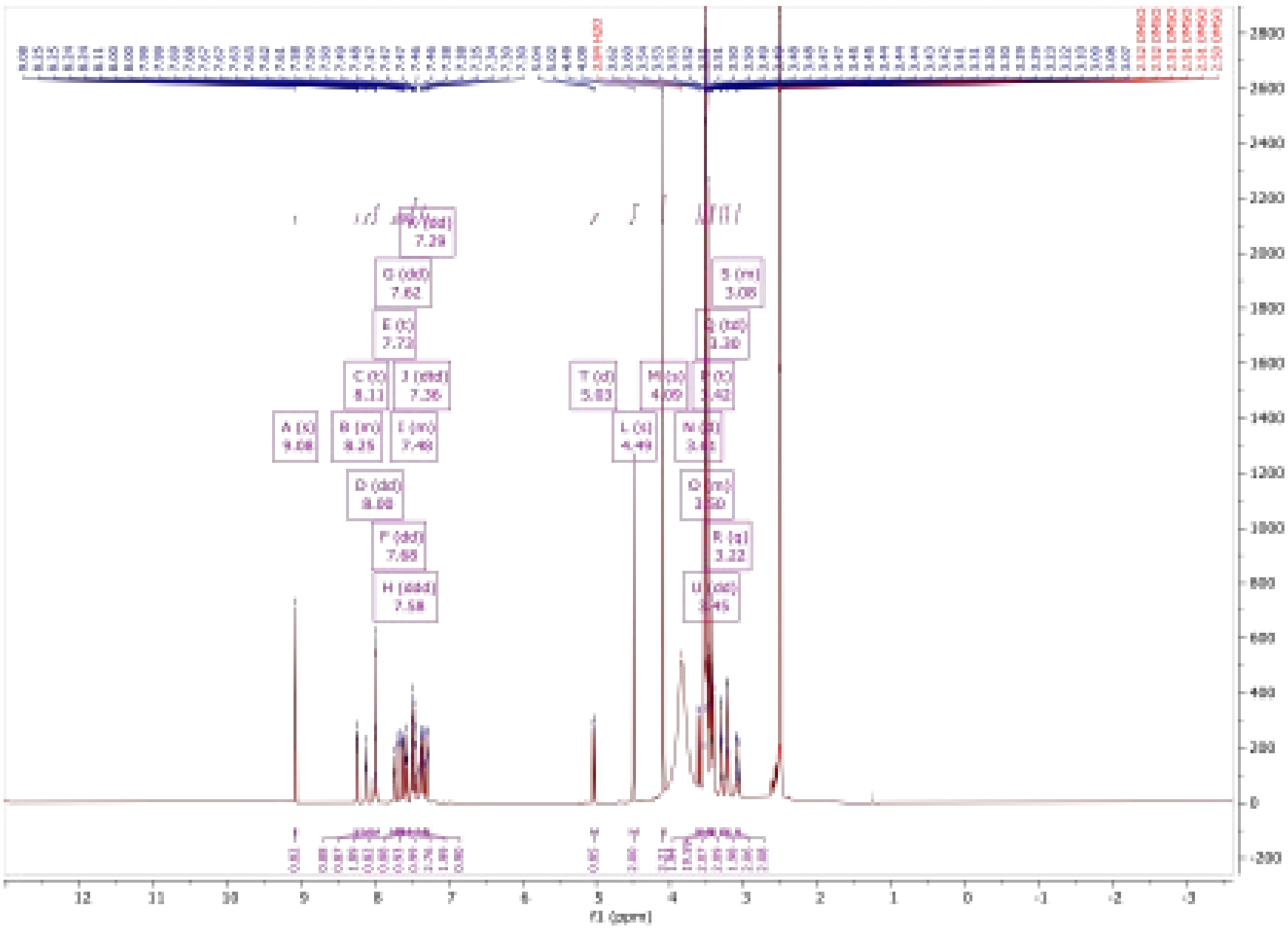

^{13}C NMR spectrum of DBCO-PEG6-dFl:

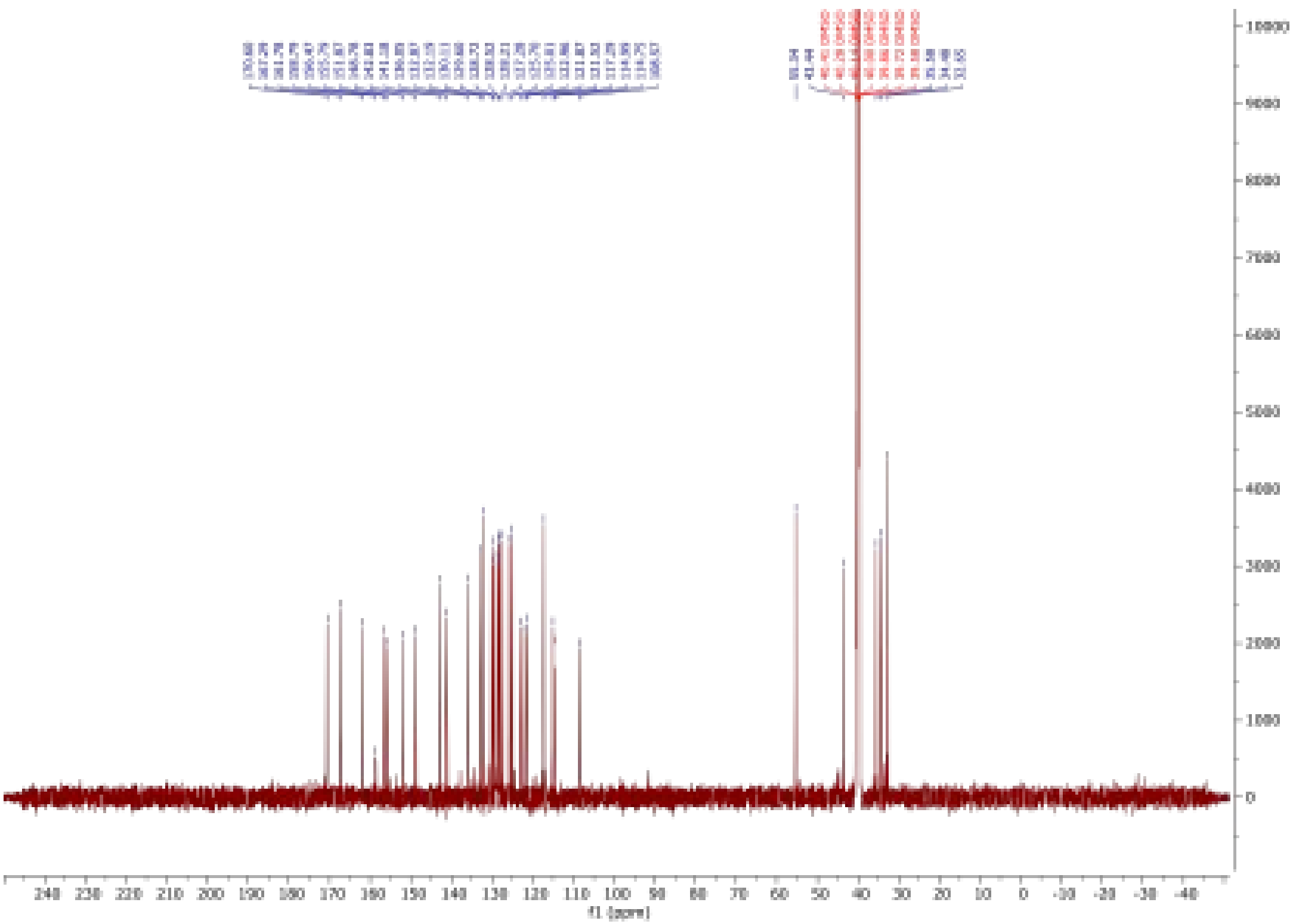

Ir-PEG6-NHBoc

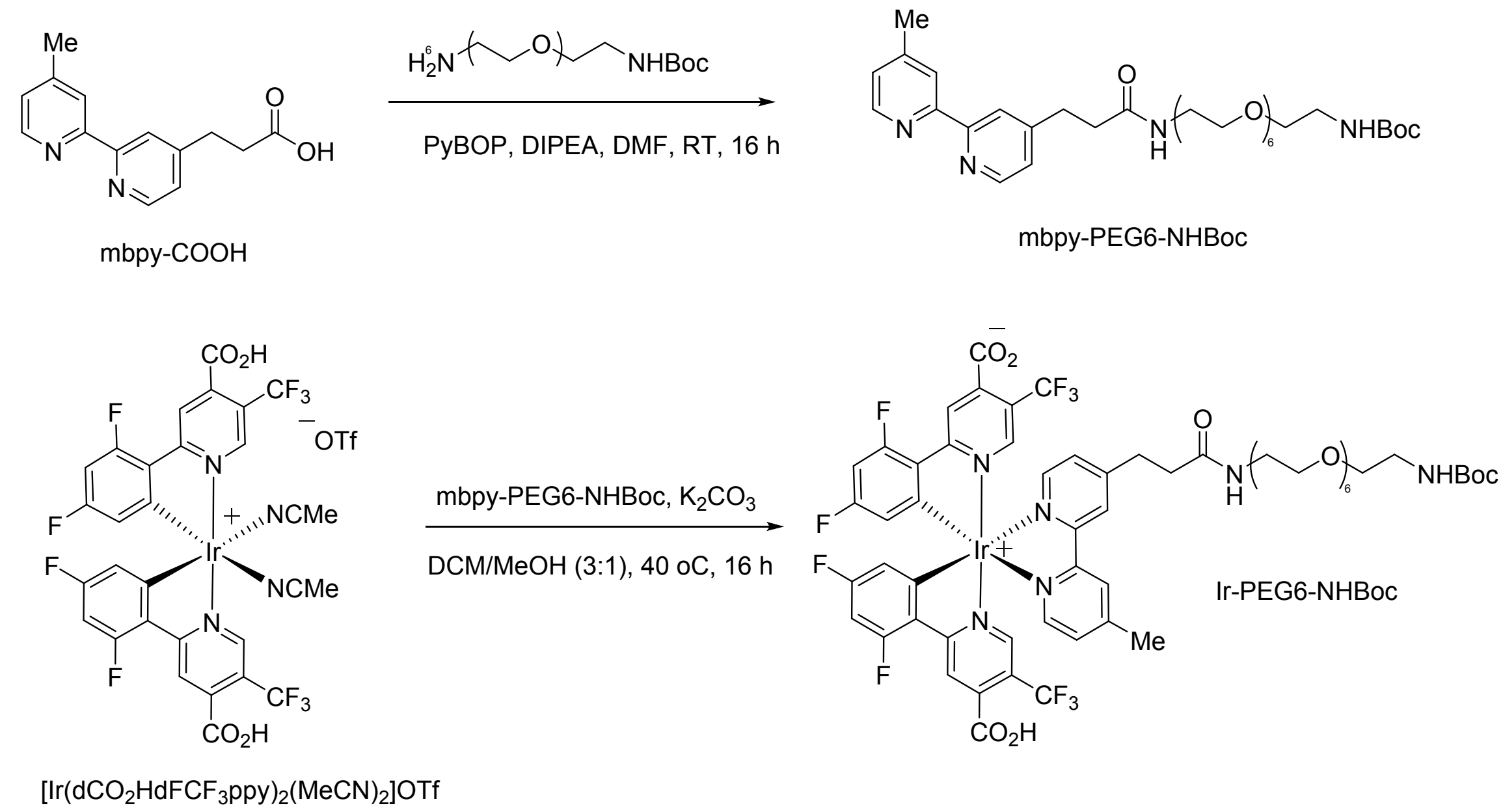

mbpy-PEG6-NHBoc:

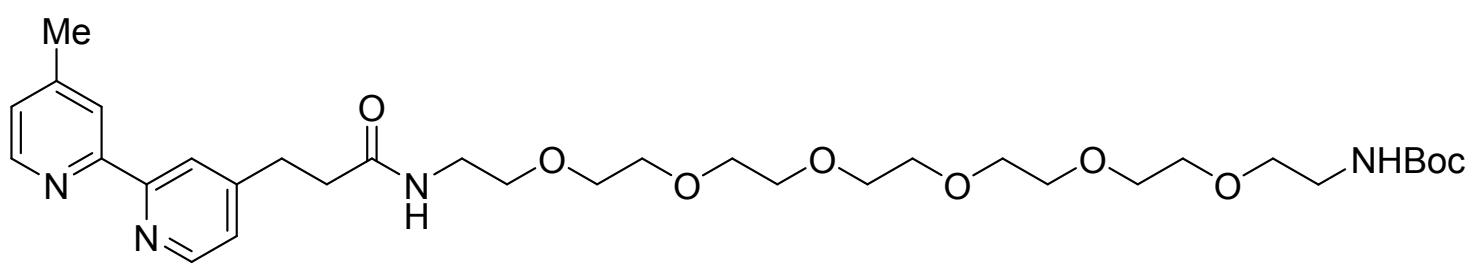

Boc-NH-PEG$_6$-NH$_2$ (254.7 mg, 0.6 mmol) dissolved in dry DMF (3 mL) was added to a solution of 3-(4'-methyl-[2,2'-bipyridin]-4-yl)propanoic acid (121.1 mg, 0.5 mmol)[8] PyBOP (312.2 mg, 0.6 mmol) and DIPEA (0.26 mL, 1.5 mmol) in dry DMF (3 mL) under Ar atmosphere. The reaction mixture was stirred for 16 h at room temperature and subsequently diluted with EtOAc (50 mL) and washed with water (25 mL), sat. NaHCO$_3$ (25 mL) and brine (25 mL). The organic layer was then dried over Na2SO4 and concentrated under reduced pressure to provide a yellow oil that was purified by silica gel column chromatography (0-10% MeOH in DCM) to provide the desired compound as a colourless oil (302 mg, 0.47 mmol, 78%).

^{1}H NMR (600 MHz, CD$_3$CN) δ = 8.65 (d, J = 5.2 Hz, 2H), 8.32 (t, J = 2.1 Hz, 2H), 7.53 – 7.45 (m, 2H), 6.81 (t, J = 5.7 Hz, 1H), 5.47 (s, 1H), 3.57 – 3.52 (m, 16H), 3.51 – 3.49 (m, 2H), 3.49 – 3.44 (m, 4H), 3.42 (t, J = 5.6 Hz, 2H), 3.29 (q, J = 5.5 Hz, 2H), 3.18 (q, J = 5.5 Hz, 2H), 3.09 (t, J = 7.4 Hz, 2H), 2.61 (t, J = 7.4 Hz, 2H), 2.56 (s, 3H), 1.42 (s, 9H) ppm.

HRMS (ESI) m/z: [M + H]$^+$ Calcd for $C_{33}H_{53}ClN_4O_9$ 649.3807; Found 649.3889

¹H NMR spectrum of mbpy-PEG6-NHBoc:

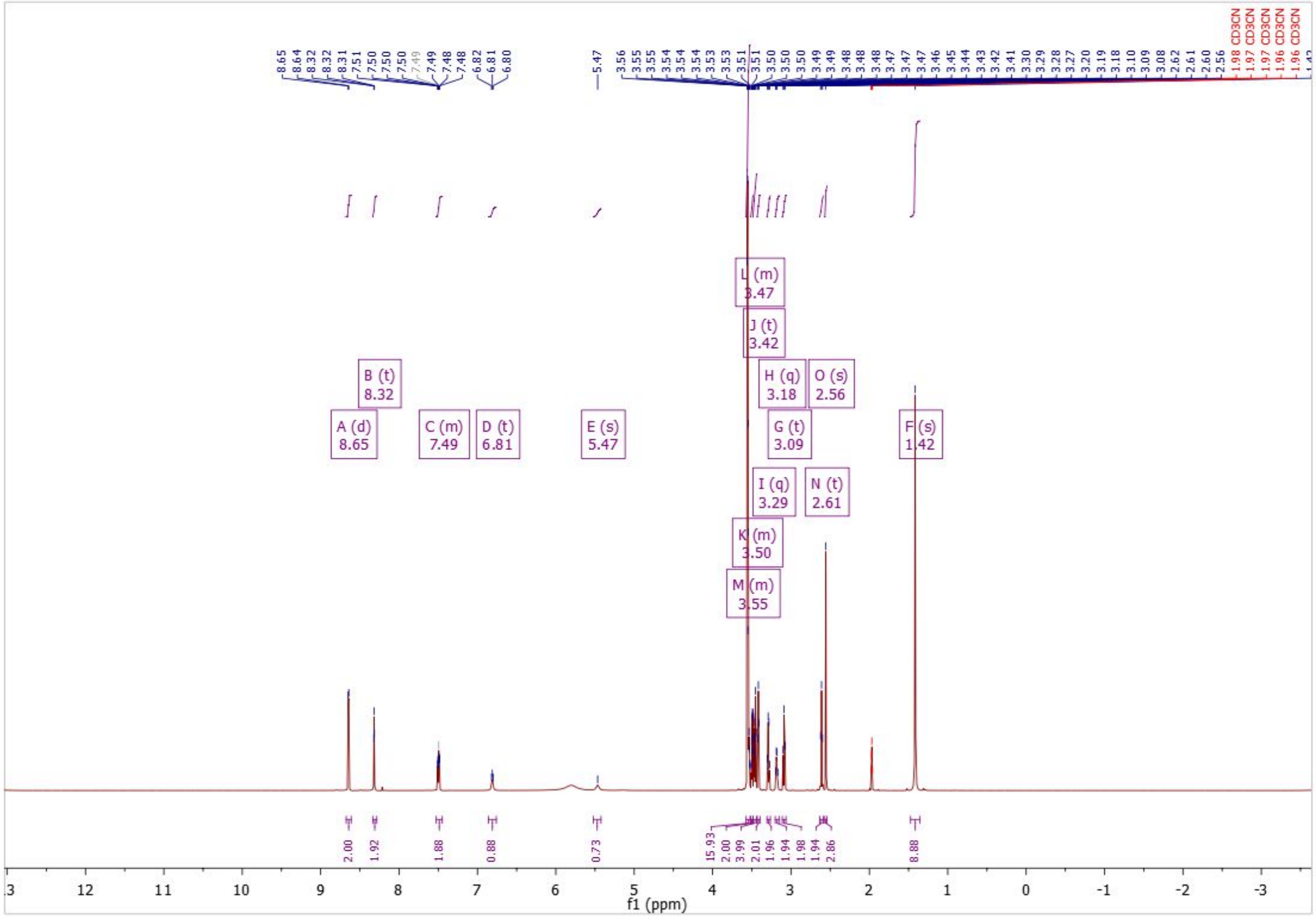

Ir-PEG6-NHBoc:

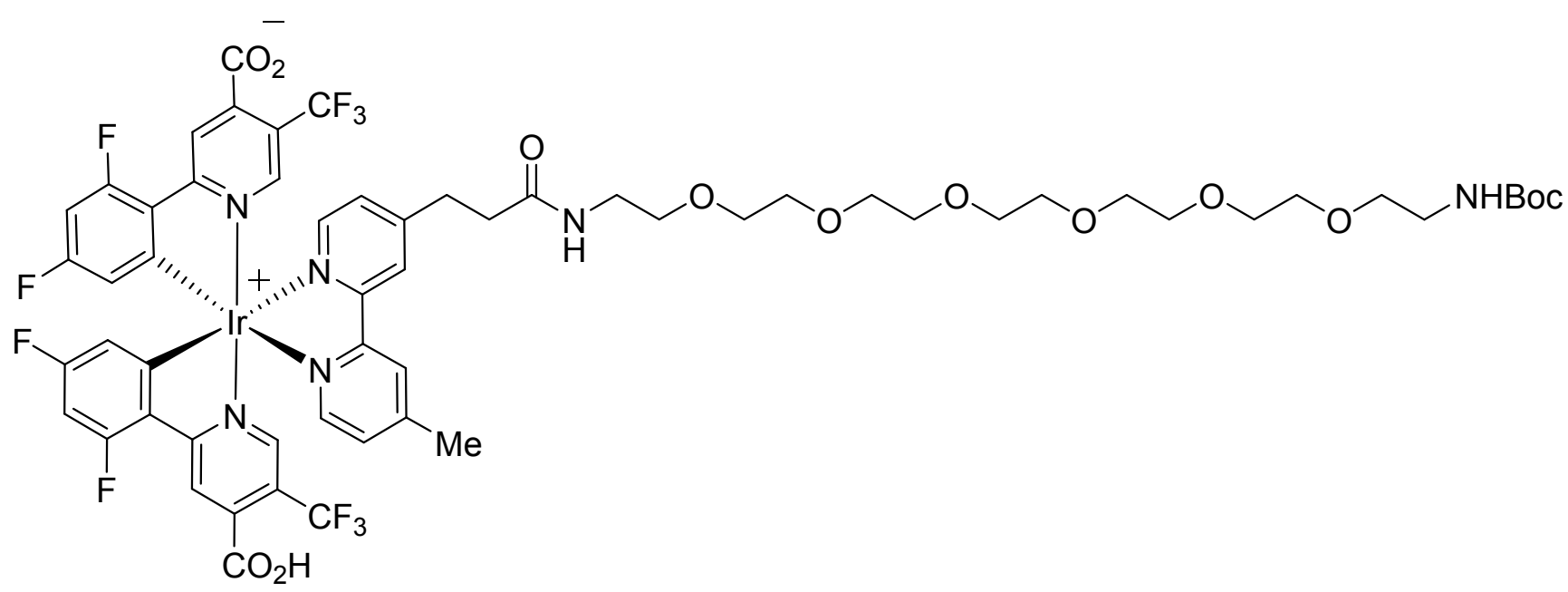

$[Ir(dCO_2HdFCF3ppy)_2(MeCN)_2]OTf$ (70.0 mg, 68.1 µmol)[9] and K_2CO_3 (13.0 mg, 94.1 µmol) were suspended in DCM/MeOH (6 mL, 3:1 v/v) and mbpy-PEG6-NHBoc (48.6 mg, 74.9 µmol) dissolved in DCM/MeOH (3 mL, 3:1 v/v) was added dropwise under Ar atmosphere**.** The resulting mixture was stirred at 40 °C for 16 hours before being concentrated under reduced pressure directly onto silica gel. The crude product was purified by flash silica gel column chromatography (0-30% MeOH in DCM) to provide the desired compound as a yellow solid (70.2 mg, 71% yield).

^{1}H NMR (600 MHz, CD_3OD) δ = 8.72 (d, *J* = 1.6 Hz, 2H), 8.40 (dd, *J* = 5.7, 2.6 Hz, 2H), 7.95 (dd, *J* = 27.1, 5.6 Hz, 2H), 7.69 – 7.53 (m, 4H), 6.80 – 6.72 (m, 2H), 5.85 (ddd, *J* = 10.9, 8.1, 2.3 Hz, 2H), 3.63 (d, *J* = 4.0 Hz, 14H), 3.62 – 3.59 (m, 4H), 3.57 (q, *J* = 5.0, 4.5 Hz, 2H), 3.50 (dt, *J* = 13.8, 5.1 Hz, 4H), 3.35 (d, *J* = 5.9 Hz, 2H), 3.22 (t, *J* = 5.7 Hz, 4H), 2.71 (t, *J* = 7.4 Hz, 2H), 2.67 (s, 3H), 1.44 (s, 9H) ppm.

^{13}C NMR (151 MHz, CD_3OD) δ = 173.7, 170.9, 168.9, 168.8, 167.0 (d, *J* = 13.2 Hz), 165.3 (d, *J* = 12.1 Hz), 164.8 (d, *J* = 15.1 Hz), 163.1 (d, *J* = 13.6 Hz), 157.7, 156.8, 156.6, 156.50 – 156.12 (m), 155.06, 152.83 – 152.11 (m), 151.7, 151.4, 147.49 – 146.70 (m), 131.0, 130.2, 127.9, 127.3, 126.7, 124.3, 122.47 (dd, *J* = 20.8, 7.3 Hz), 115.2 (t, *J* = 18.1 Hz), 100.5 (t, *J* = 27.2 Hz), 80.1, 71.5 – 71.4 (m, 8C), 71.2, 71.1 (2C), 70.5, 49.43, 49.28, 49.14, 49.00, 48.86, 48.72, 48.57, 41.27, 40.34, 36.22, 31.96, 28.78, 21.49 ppm.

HRMS (ESI) m/z: $[M]^+$ Calcd for $IrC_{59}H_{61}F_{10}N6O_{13}$ 1444.3766; Found 1444.3727

^{1}H NMR of Ir-PEG6-NHBoc:

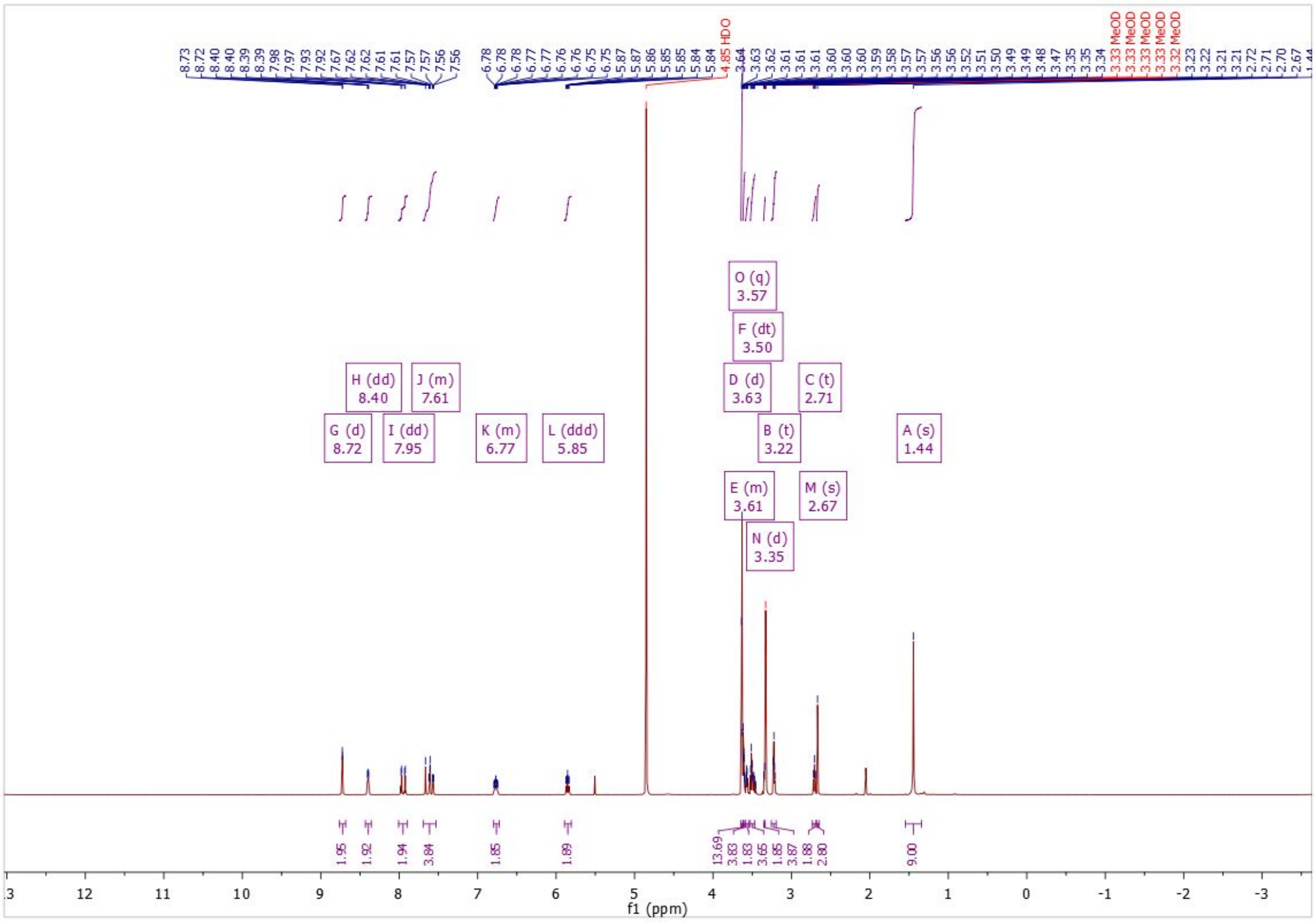

^{13}C NMR of Ir-PEG6-NHBoc:

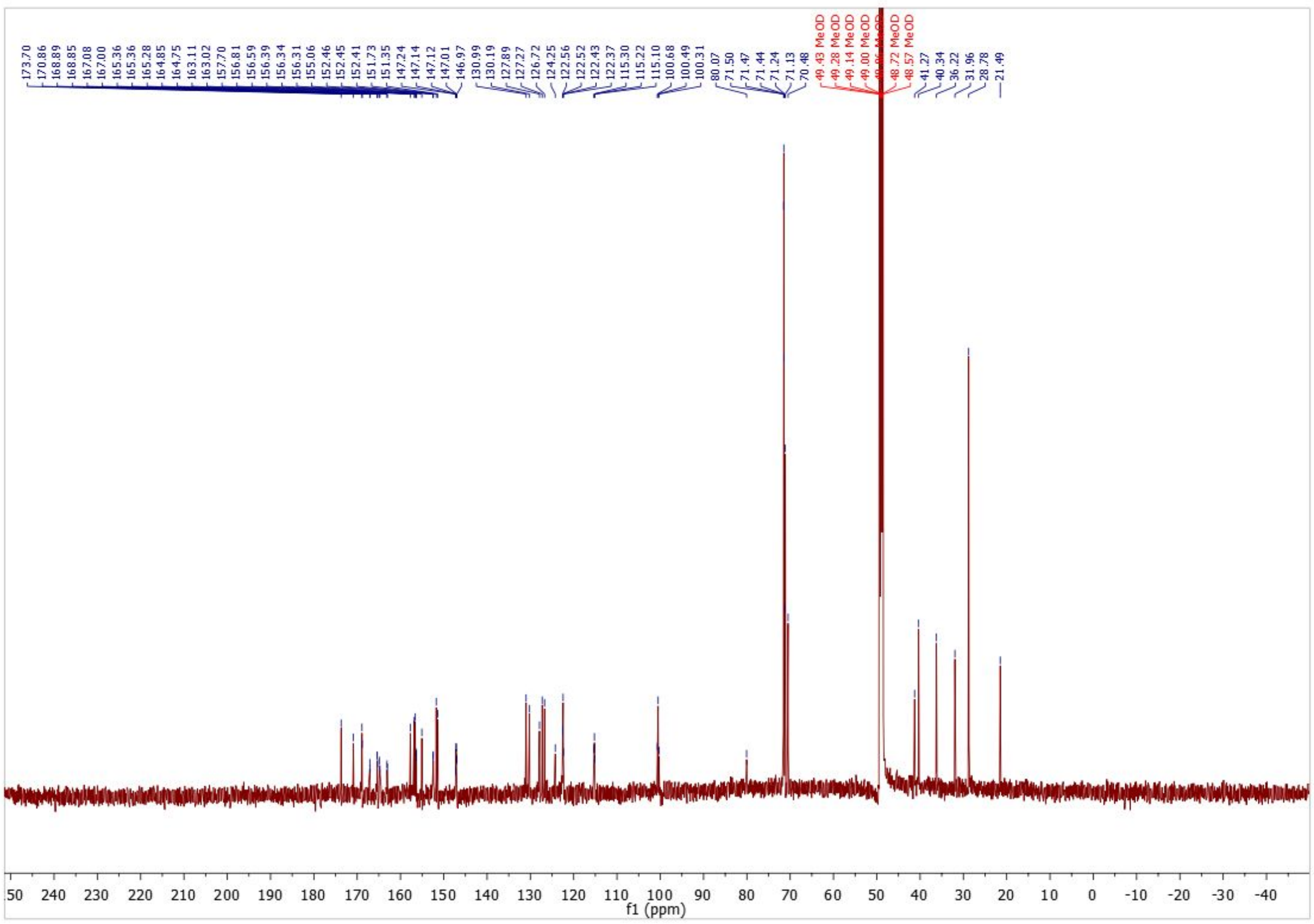

Ir-PEG6-DBCO

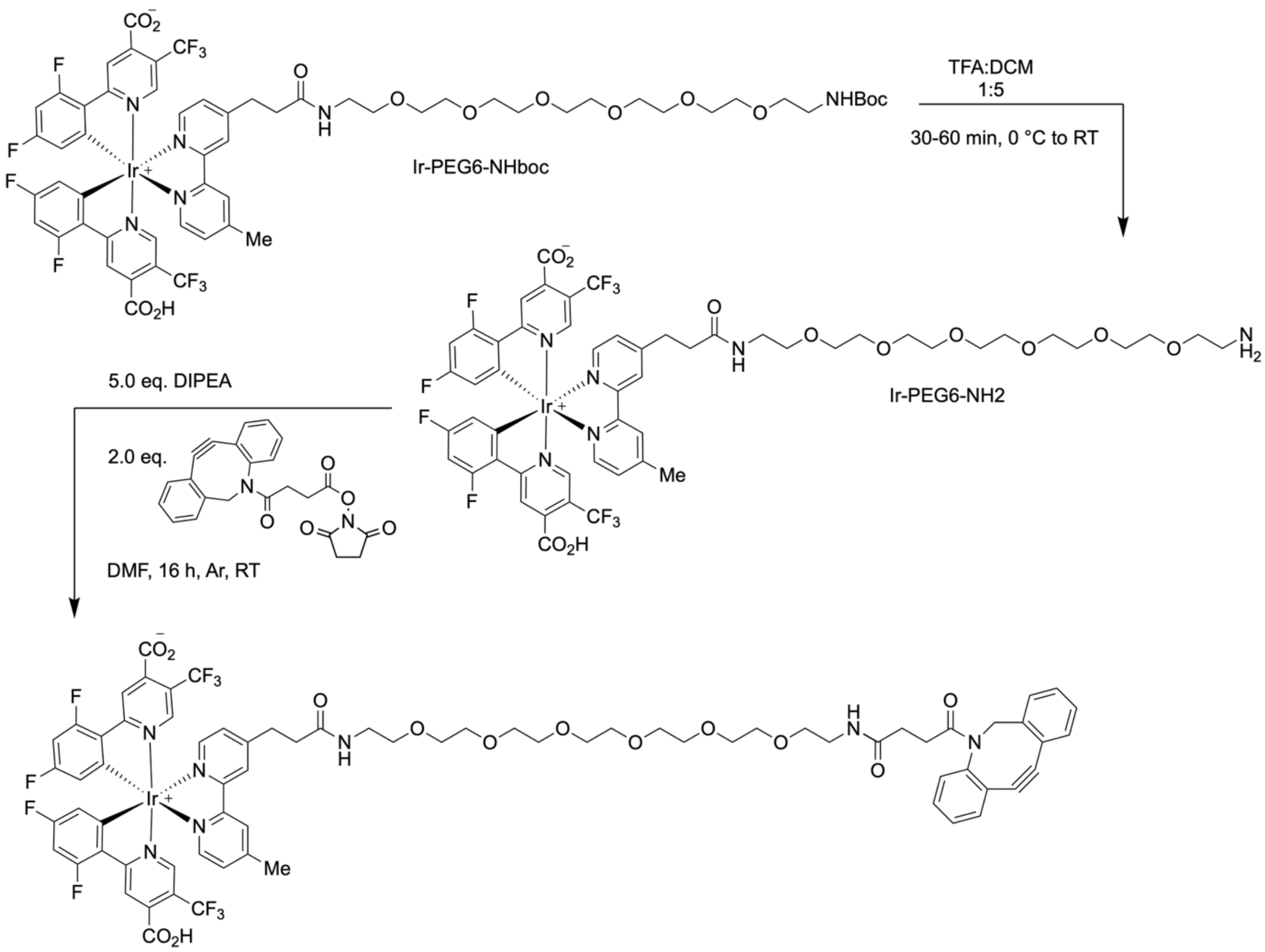

Ir-PEG6-DBCO was synthesised analogous to a published protocol.[10] Ir-PEG6-NHBoc (20 mg, 13.8 µmol) was added to a brown 10 mL vial and dissolved in 500 µL DCM. The flask was cooled to 0 °C before the adding 100 µL TFA. Boc-deprotection was carried out by warming the reaction mixture to room temperature and stirring for 30-60 minutes. The completed reaction was concentrated under reduced pressure and MeOH was added to remove excess acid from the solid, before it was concentrated under reduced pressure again. This was repeated three times. The resulting Ir-PEG6-amine was dissolved in 500 µL dry DMF and 5 eq. of DIPEA were added. To this solution, 2 eq. DBCO-NHS ester (11.1 mg, 27.7 µmol) were added, and the coupling was carried out overnight under Ar atmosphere at room temperature. The desired product was purified via flash column chromatography (MeOH in DCM) and obtained as a fine yellow powder (17.9 mg, 11 µmol, 79%).

^{1}H NMR (600 MHz, DMSO) δ = 8.81 (s, 1H), 8.13 (d, J = 10.2 Hz, 1H), 7.99 (d, J = 5.7 Hz, 1H), 7.83 (dd, J = 8.8, 5.9 Hz, 1H), 7.78 (d, J = 5.8 Hz, 1H), 7.68 (d, J = 7.6 Hz, 1H), 7.64 – 7.55 (m, 2H), 7.52 – 7.43 (m, 2H), 7.37 (s, 1H), 7.34 (t, J = 7.5 Hz, 1H), 7.29 (d, J = 7.4 Hz, 1H), 7.03 (t, J = 11.1 Hz, 1H), 5.75 (t, J = 9.2 Hz, 1H), 5.04 (d, J = 14.0 Hz, 1H), 3.54 (s, 2H), 3.54 – 3.36 (m, 20H), 3.29 (d, J = 5.9 Hz, 2H), 3.18 (d, J = 6.0 Hz, 2H), 3.07 (dt, J = 15.6, 7.0 Hz, 2H), 2.60 (s, 2H), 2.56 (s, 3H), 2.28 – 2.00 (m, 1H), 1.99 (d, J = 7.0 Hz, 1H).

^{13}C NMR (151 MHz, DMSO) δ = 173.22, 171.58, 171.52, 170.94, 166.33, 165.54, 164.77, 163.16, 163.08, 162.77, 161.12, 161.03, 156.42, 155.65, 155.35, 155.26, 154.53, 153.14,

152.08, 150.45, 150.19, 148.91, 146.00, 132.88, 130.24, 130.08, 129.38, 128.59, 128.44, 128.13, 127.25, 127.17, 126.29, 125.61, 123.01, 121.89, 121.55, 121.43, 120.52, 120.30, 114.69, 114.15, 108.62, 100.08, 99.90, 99.72, 70.22, 70.15, 69.99, 69.53, 69.44, 55.36, 40.54, 40.42, 40.28, 40.15, 40.01, 39.87, 39.73, 39.59, 38.99, 38.95, 35.22, 30.96, 30.82, 30.19, 25.69, 21.41.

HR-MS (ESI$^+$) for $C_{73}H_{67}F_{10}IrN_7O_{13}^+$ $[M]^+$ calculated: 1632.4261 observed: 1632.4238; $[M+H]^{2+}$ calculated: 816.7167 observed: 816.7280.

^{1}H NMR spectrum of Ir-PEG6-DBCO:

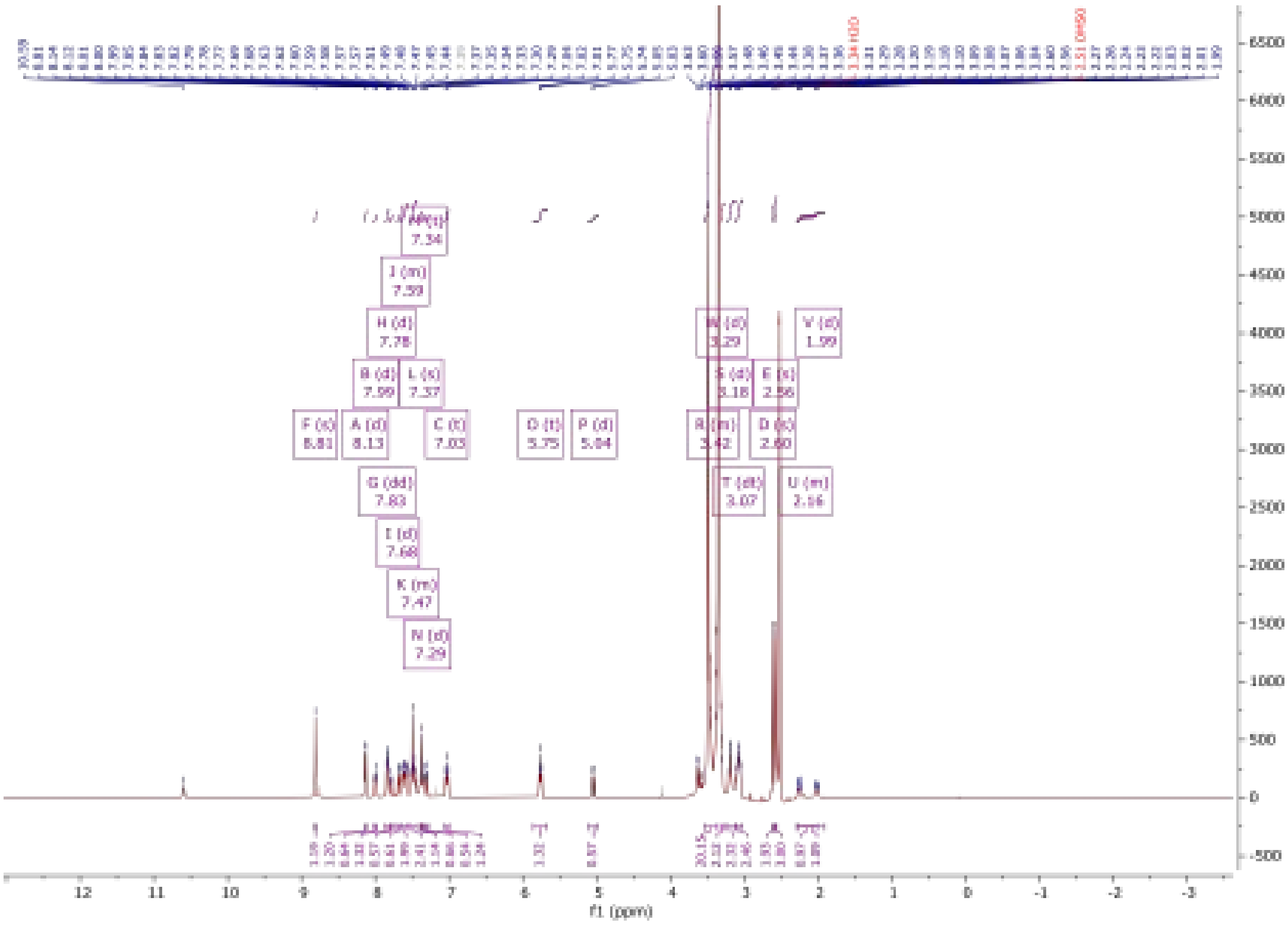

^{13}C NMR spectrum of Ir-PEG6-DBCO:

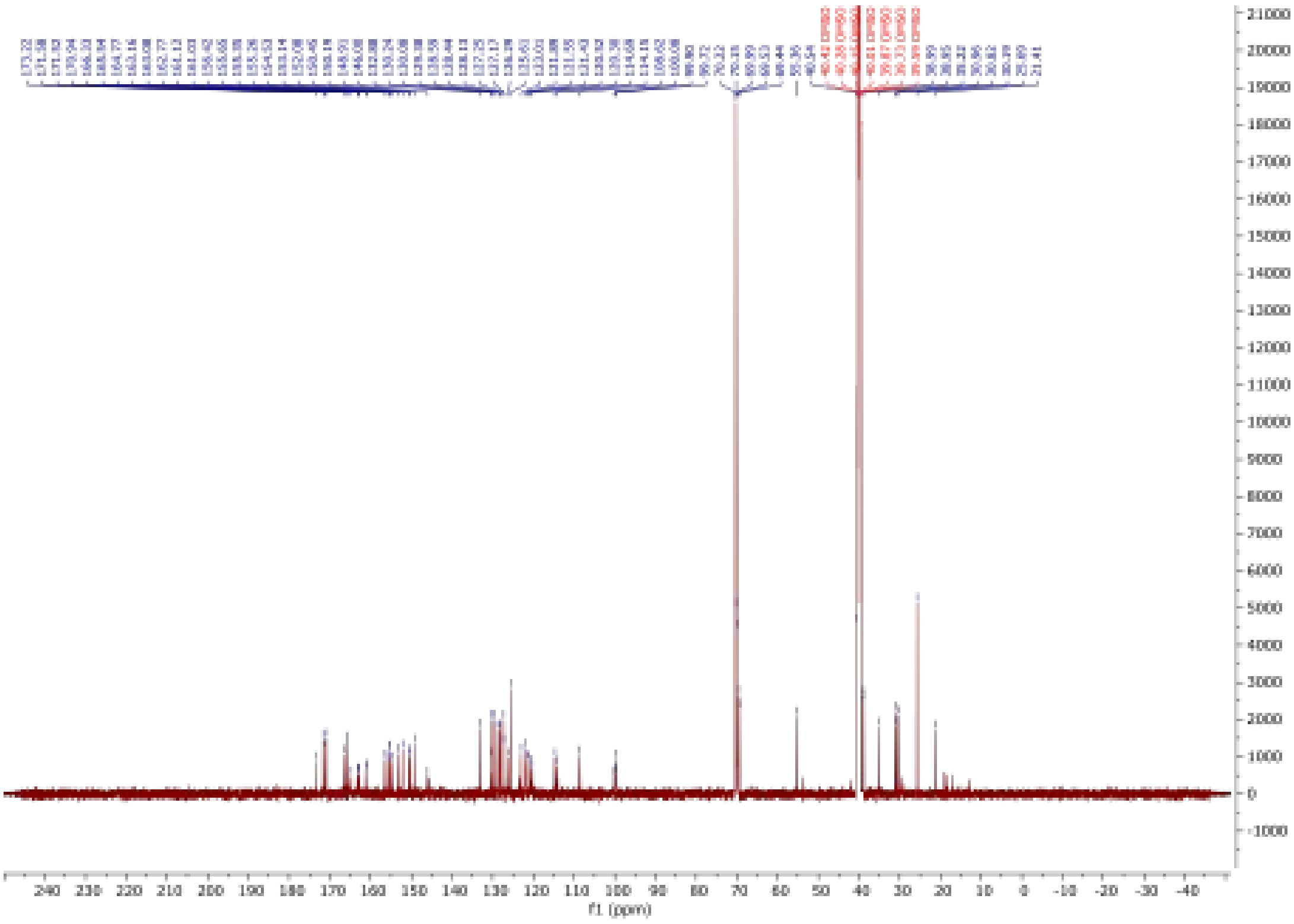

DBCO-bis-dFl

The DBCO-bis-dFl construct was synthesised by standard fluorenylmethoxycarbonyl (Fmoc)-solid-phase peptide synthesis (SPPS) on Rink amide resin (0.2 mmol scale, 0.22 mmol/g), up until the final coupling with dibenzocyclooctyne-amine.

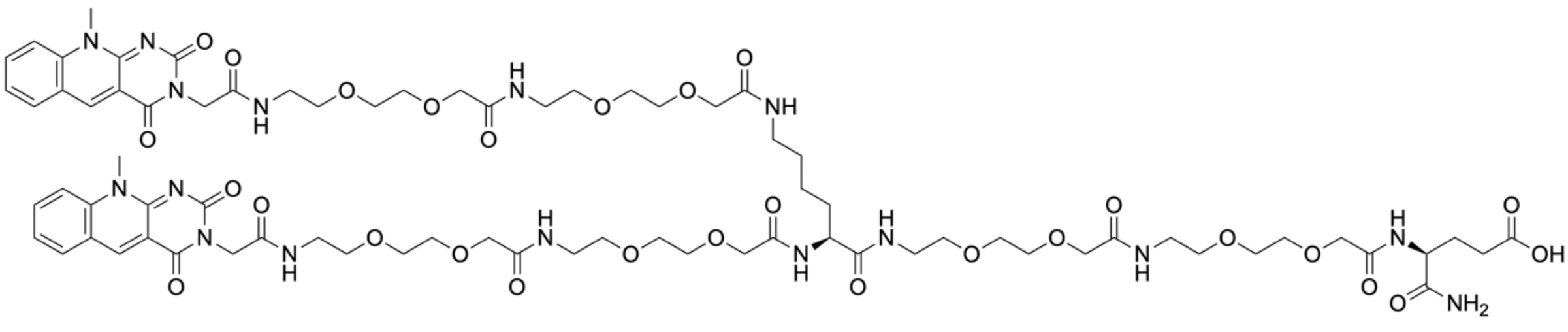

bis-dFl-Glu

The amino acids that were used to achieve a branched structure were C-terminal glutamic acid Fmoc-Glu(OtBu)-OH for successive DBCO coupling, Fmoc-8-amino-3,6-dioxaoctanoic acid for PEG2 spacer insertion and *N*α,*N*ε-di-Fmoc-L-lysine to enable parallel amino acid coupling at the *N*α as well as *N*ε amine. Amino acids were coupled in the following sequence (C- to N-terminus) using 5 eq. of amino acid with 5 eq. of HATU with 10 eq. of DIPEA in DMF, incubating for 1 h at room temperature in the dark:

Glu → PEG2 → PEG2 → Lys → 2x PEG2 → 2x PEG2

N-terminal deazaflavin conjugation was achieved using 3 eq. of dFl-COOH and HATU with 5 eq. of DIPEA in DMF for 2 h at room temperature in the dark. Final deprotection and cleavage from the resin was performed using 95% TFA, 2.5% TIS and 2.5% H_2O for 4 h at room temperature in the dark. Crude peptides were precipitated in ice-cold diethylether and purified by preparative HPLC (80 mg, 0.048 mmol, 24 %).

UPLC-UV (220 nm) trace:

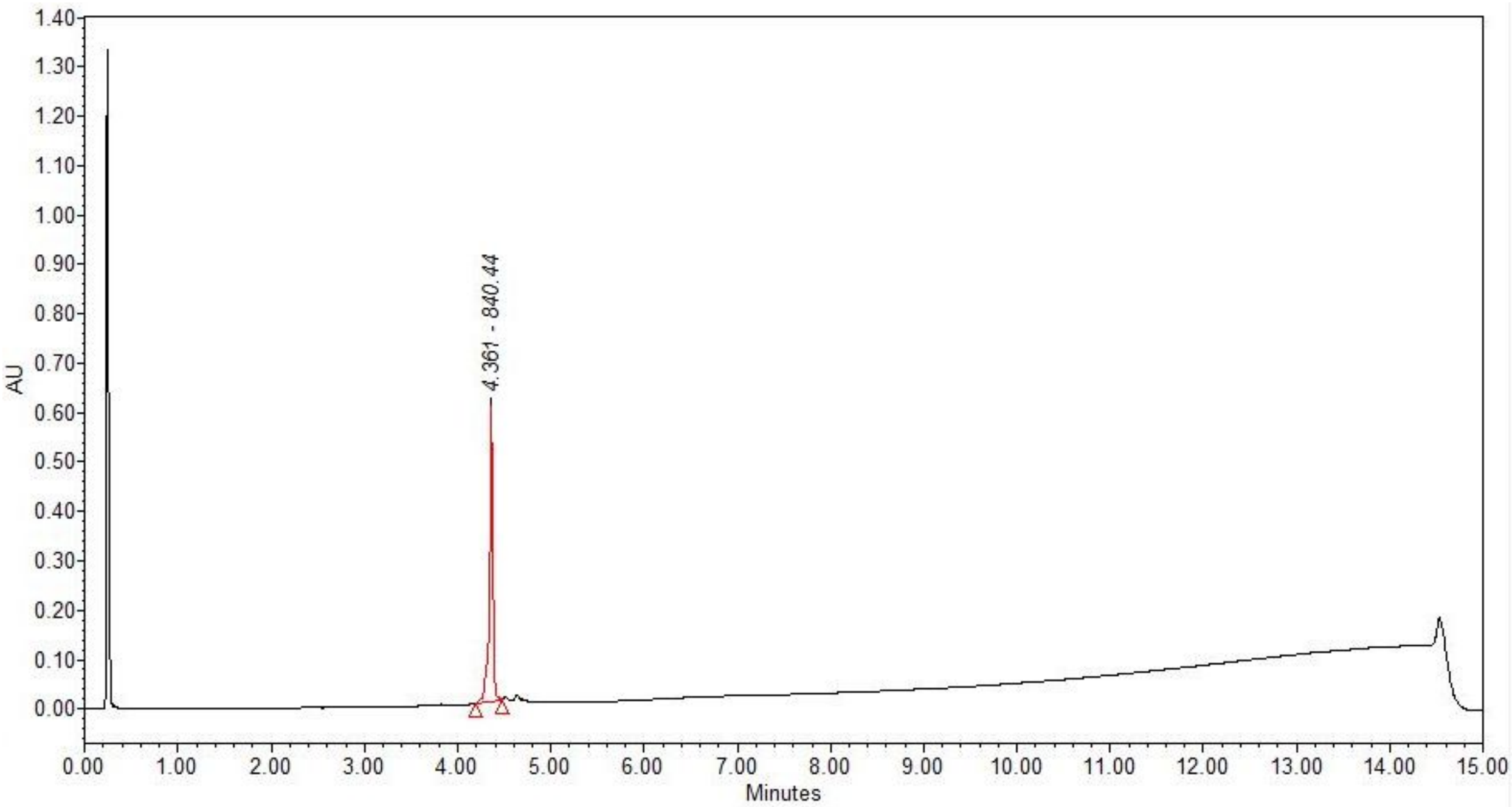

HR-MS (ESI$^+$) for $C_{75}H_{106}N_{16}O_{28}$ $[M+3H]^{3+}$ calculated: 560.5860 observed: 560.5836.

Coupling with DBCO-amine:

To equip the bis-dFl linker with a DBCO moiety, the C-terminal glutamic acid derivative bis-dFl-Glu (20 mg, 0.012 mmol), dibenzocyclooctyne-amine (1.5 eq., 4.9 mg, 0.018 mmol) and benzotriazol-1-yloxytripyrrolidinophosphonium hexafluorophosphate (PyBOP; 1.2 eq., 7.5 mg, 0.014 mmol). were dissolved in 170 µL DMF each. The solutions of bis-dFl-Glu and PyBOP were mixed and 5 eq. DIPEA (10 µL, 7.8 mg, 0.06 mmol) were added. Finally, the dibenzocyclooctyne-amine solution was added and the reaction mixture incubated for 1 h at room temperature. Afterwards, the reaction was diluted with ACN and water and subsequently subjected to preparative HPLC purification (11.6 mg, 0.006 mmol, 50 %).

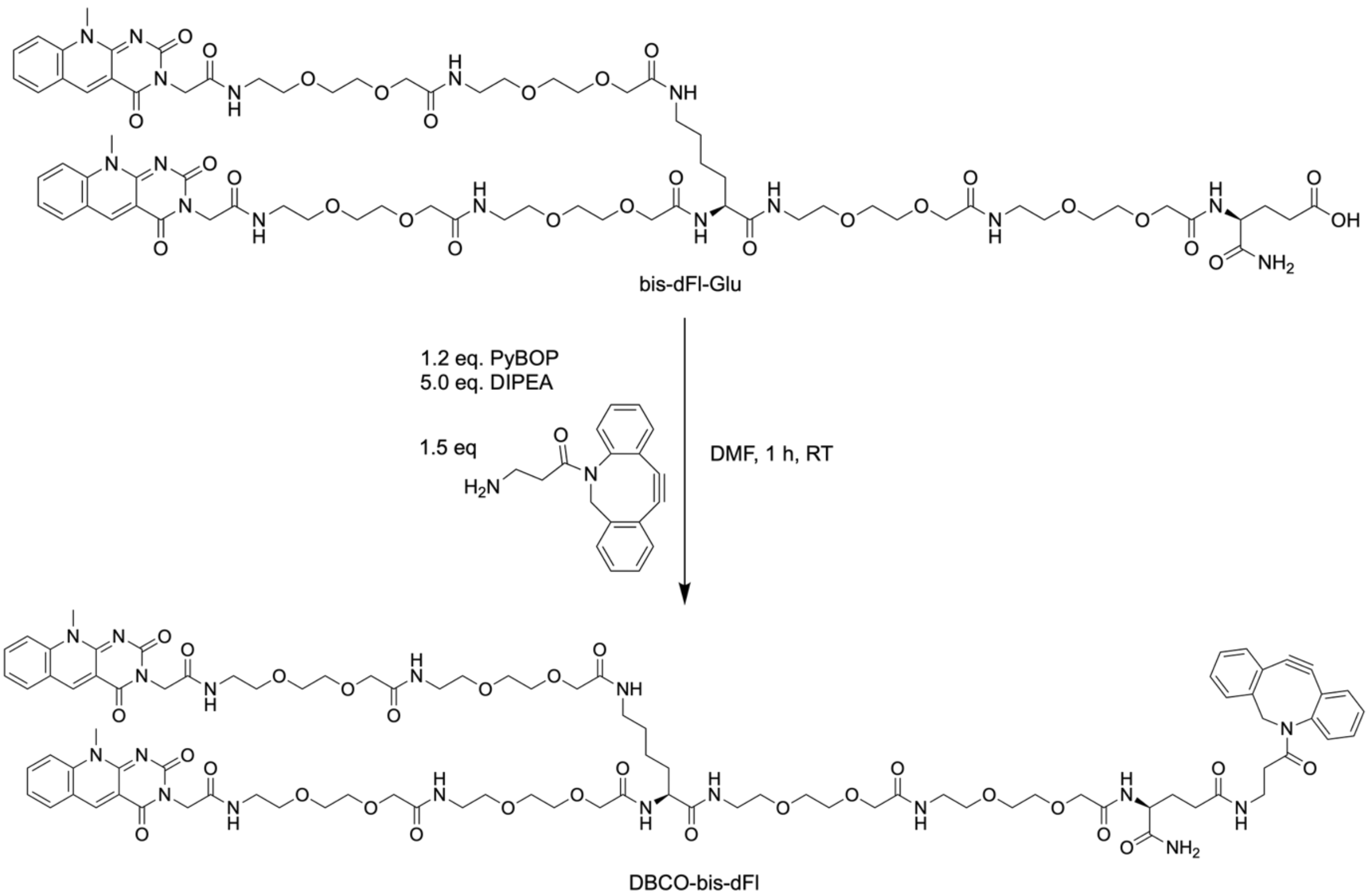

UPLC-UV (220 nm) trace:

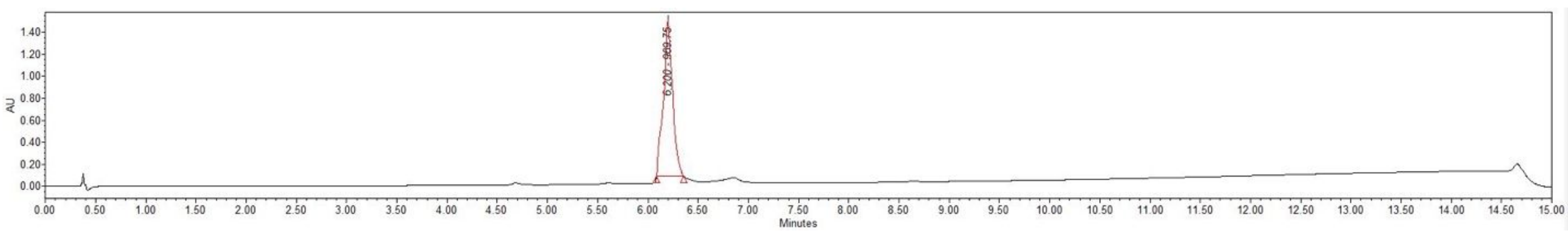

HR-MS (ESI$^+$) for $C_{93}H_{120}N_{18}O_{28}$ $[M+2H]^{2+}$ calculated: 969.9402 observed: 969.9492; $[M+3H]^{3+}$ calculated: 646.9633 observed: 646.9636.

Diazirine-PEG4-biotin

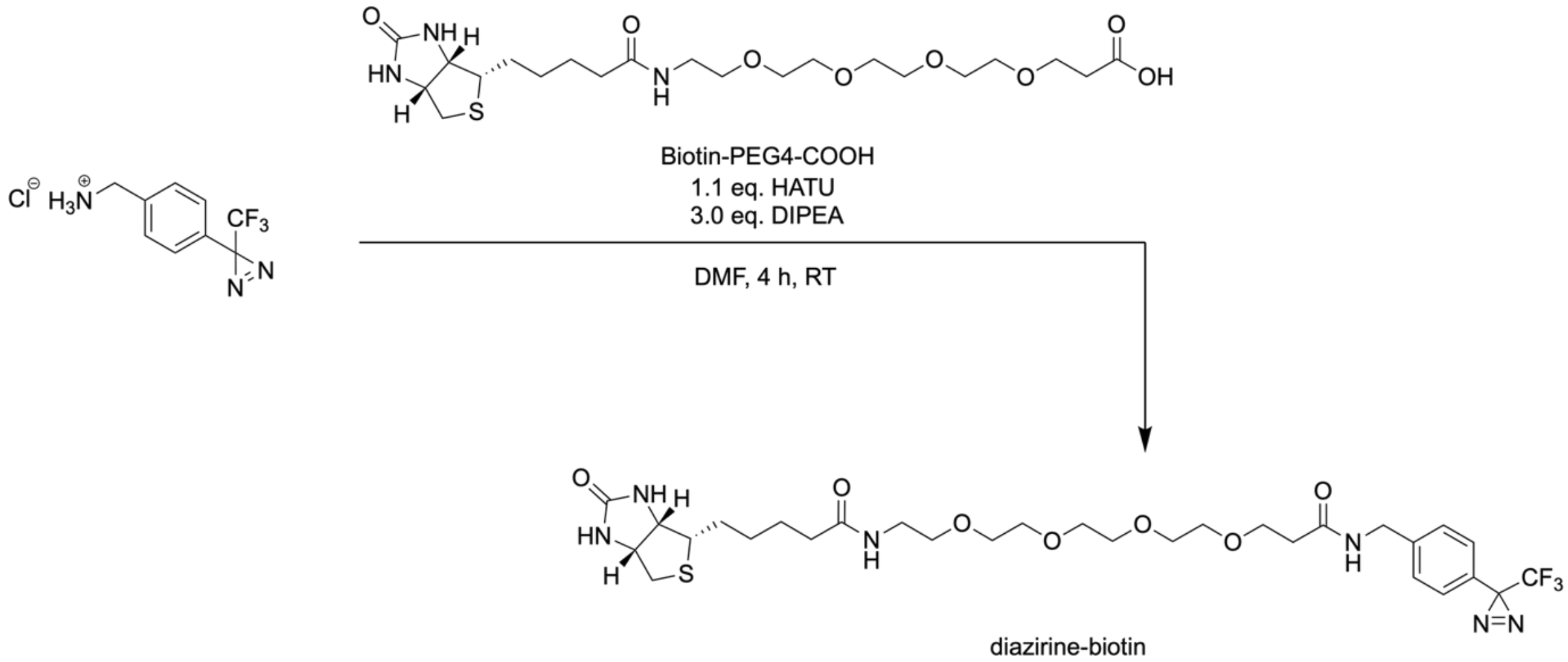

A solution of Biotin-PEG4-COOH (1 eq., 30 mg, 60 µmol) and HATU (1.1 eq., 24 mg, 66 µmol) in 700 µL DMF was prepared. Then, 31 µL DIPEA (3 eq., 21.7 mg, 180 µmol) were added and immediately afterwards a solution of 14.3 mg 4-[3-(trifluoromethyl)-3H-diazirin-3-yl]benzylamine hydrochloride (1 eq., 60 µmol) in 200 µL DMF. The reaction mixture was stirred at room temperature for 4 h in the dark. The product was purified by preparative HPLC, lyophilized and obtained as a white solid (32.4 mg, 46.8 µmol, 78%).

^{1}H NMR (600 MHz, DMSO) δ = 8.68 (t, *J* = 5.6 Hz, 1H), 8.00 – 7.91 (m, 2H), 7.81 (t, *J* = 5.7 Hz, 1H), 7.37 (d, *J* = 8.2 Hz, 2H), 4.31 – 4.30 (m, 1H), 4.14 – 4.12 (m, 1H), 3.57 – 3.52 (m, 5H), 3.51 (dd, *J* = 5.6, 3.1 Hz, 3H), 3.41 (dt, *J* = 24.4, 5.8 Hz, 5H), 3.19 – 3.16 (m, 2H), 3.11 – 3.07 (m, 1H), 2.83 – 2.80 (m, 1H), 2.58 (d, *J* = 12.4 Hz, 1H), 2.06 (t, *J* = 7.4 Hz, 2H), 1.61 (ddt, *J* = 13.6, 9.8, 6.1 Hz, 1H), 1.47 (dddd, *J* = 23.0, 15.0, 11.4, 7.9 Hz, 3H), 1.30 (dddd, *J* = 19.1, 9.5, 5.9, 2.6 Hz, 2H).

^{13}C NMR (151 MHz, DMSO) δ = 172.64, 165.72, 163.22, 159.17, 158.92, 158.67, 158.42, 136.43, 130.66, 128.57, 126.86, 124.96, 123.14, 121.32, 119.49, 116.63, 114.71, 70.03, 70.00, 69.62, 69.24, 61.53, 59.70, 55.88, 40.50, 40.38, 40.29, 40.24, 40.11, 39.97, 39.83, 39.76, 39.69, 39.55, 38.90, 35.56, 28.90, 28.64, 28.49, 28.37, 28.10, 25.71.

^{19}F NMR (564 MHz, None) δ = -64.25.

HR-MS (ESI^{+}) for $C_{30}H_{43}F_{3}N_{6}O_{7}S$ $[M+H]^{+}$ calculated: 689.2939 observed: 689.2947.

^{1}H NMR spectrum of diazirine biotin:

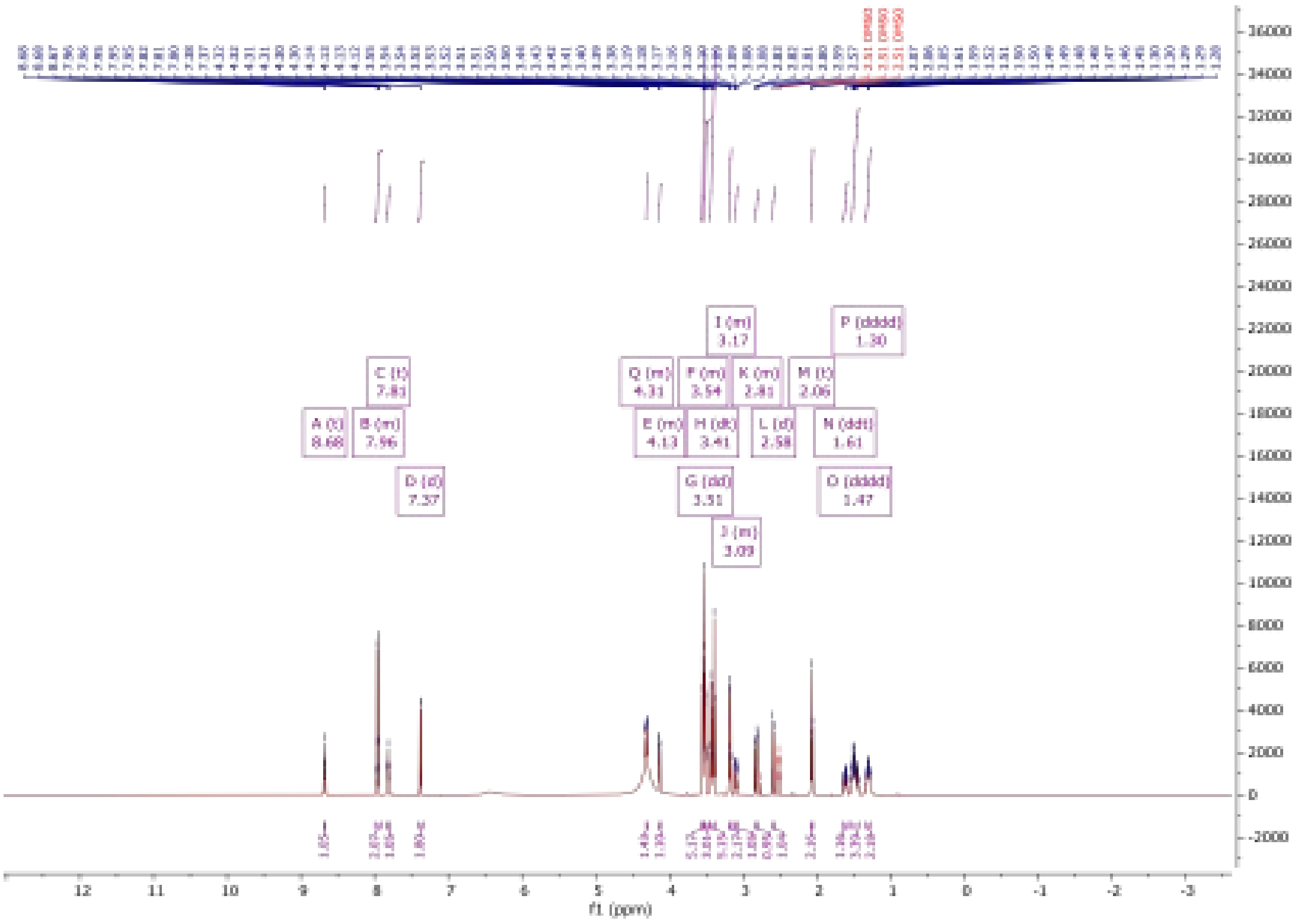

^{13}C NMR spectrum of diazirine-biotin:

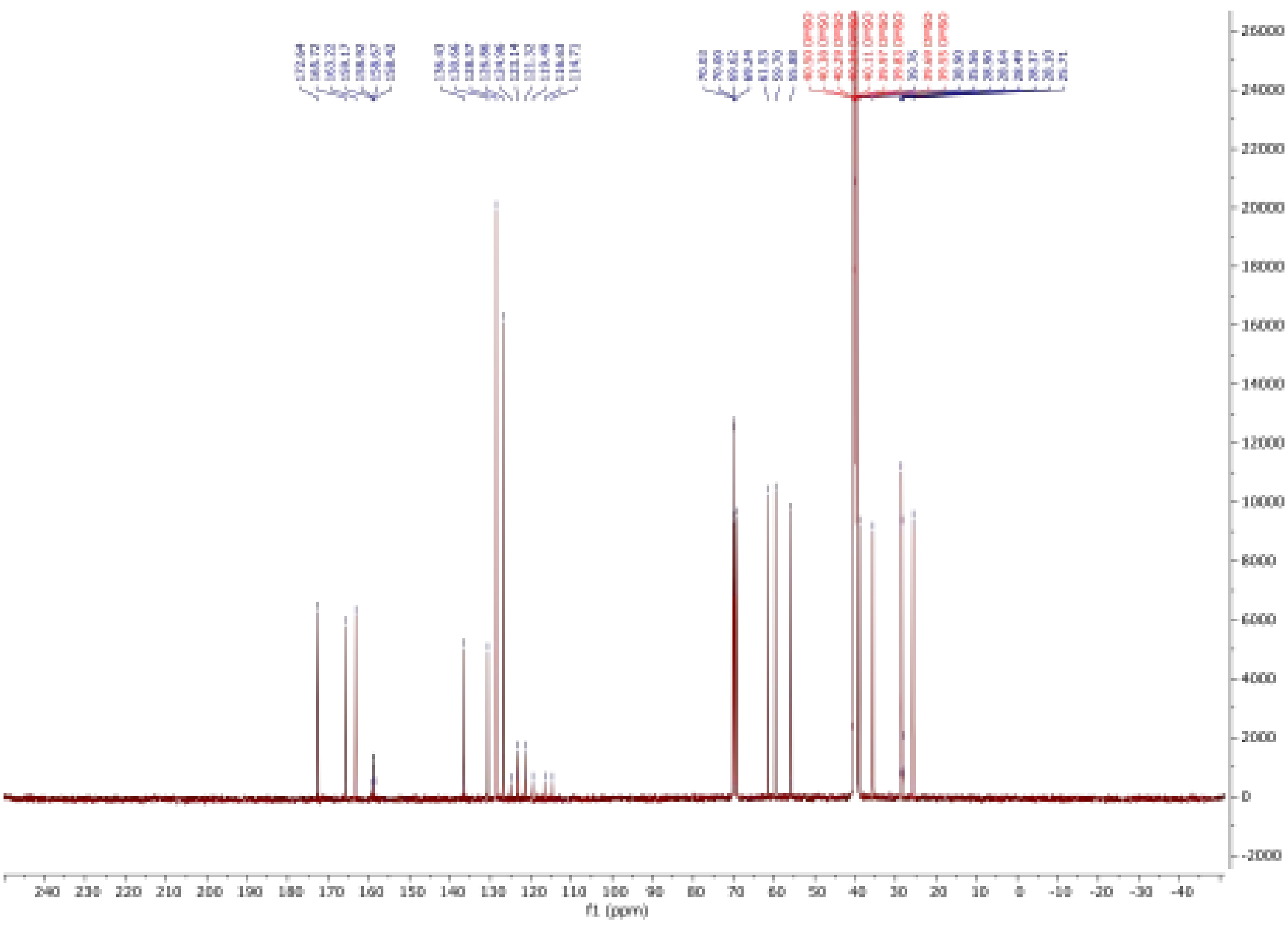

^{19}F NMR spectrum of diazirine biotin:

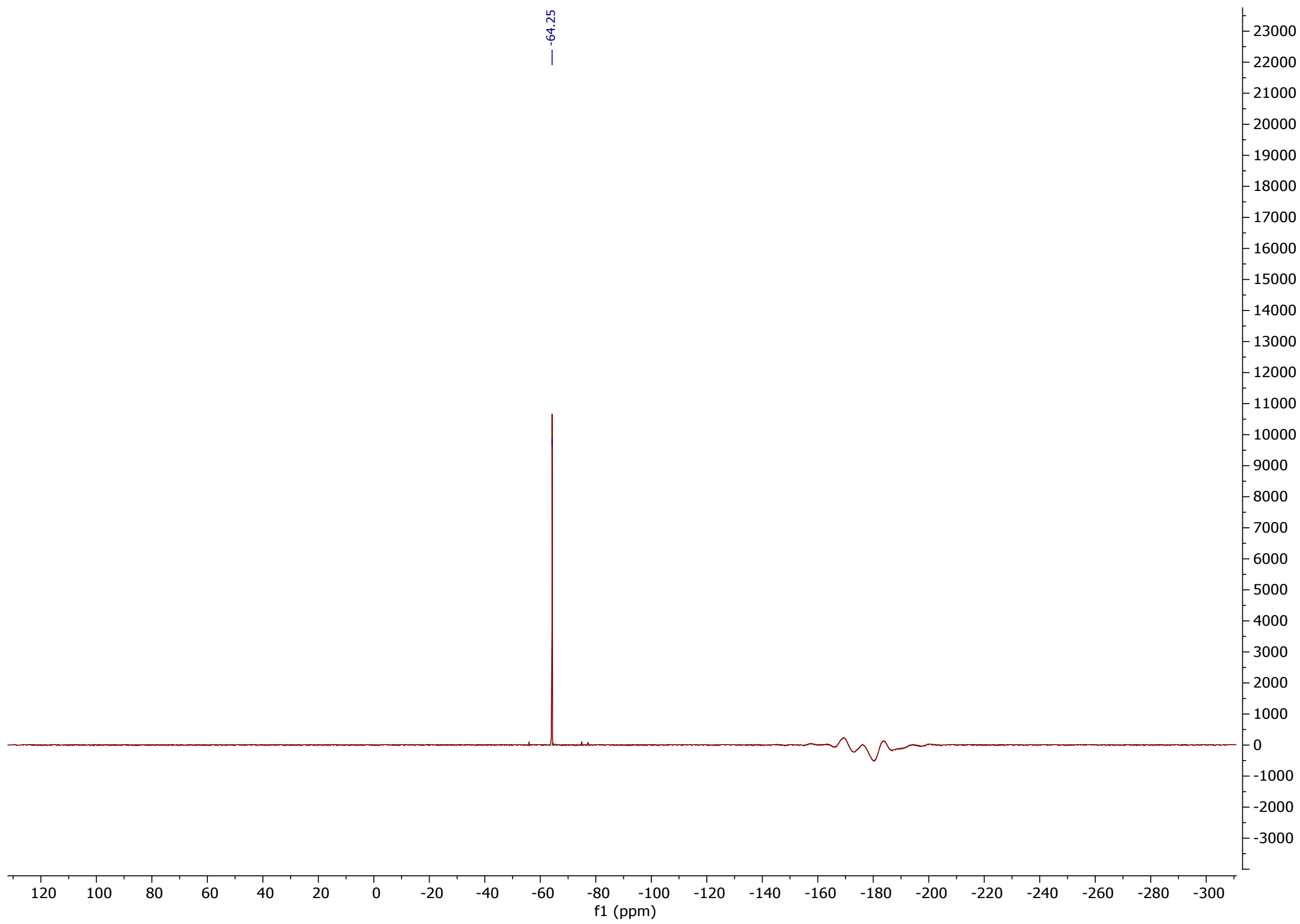

References

(1) Kong, A. T.; Leprevost, F. V.; Avtonomov, D. M.; Mellacheruvu, D.; Nesvizhskii, A. I. MSFragger: ultrafast and comprehensive peptide identification in mass spectrometry-based proteomics. *Nat Methods* **2017**, *14* (5), 513-520.

(2) The, M.; MacCoss, M. J.; Noble, W. S.; Kall, L. Fast and Accurate Protein False Discovery Rates on Large-Scale Proteomics Data Sets with Percolator 3.0. *J Am Soc Mass Spectrom* **2016**, *27* (11), 1719-1727.

(3) Keller, A.; Nesvizhskii, A. I.; Kolker, E.; Aebersold, R. Empirical Statistical Model To Estimate the Accuracy of Peptide Identifications Made by MS/MS and Database Search. *Anal Chem* **2002**, *74* (20), 5383-5392.

(4) Yu, F.; Haynes, S. E.; Nesvizhskii, A. I. IonQuant Enables Accurate and Sensitive Label-Free Quantification With FDR-Controlled Match-Between-Runs. *Mol Cell Proteomics* **2021**, *20*, 100077.

(5) Hsiao, Y.; Zhang, H.; Li, G. X.; Deng, Y.; Yu, F.; Valipour Kahrood, H.; Steele, J. R.; Schittenhelm, R. B.; Nesvizhskii, A. I. Analysis and Visualization of Quantitative Proteomics Data Using FragPipe-Analyst. *J Proteome Res* **2024**, *23* (10), 4303-4315.

(6) Ge, S. X.; Jung, D.; Yao, R. ShinyGO: a graphical gene-set enrichment tool for animals and plants. *Bioinformatics* **2020**, *36* (8), 2628-2629.

(7) Crocker, L. B.; Arafiles, J. V. V.; Muchler, J. M.; Ruwolt, M.; Kemnitz-Hassanin, K.; Rossmann, K.; Stieger, C. E.; Liu, F.; Archipowa, N.; Kutta, R. J.; et al. Energy-transfer

photoproximity labelling in live cells using an organic cofactor. *Nat Chem* **2025**, *17* (12), 1928-1940.
(8) Trowbridge, A. D.; Seath, C. P.; Rodriguez-Rivera, F. P.; Li, B. X.; Dul, B. E.; Schwaid, A. G.; Buksh, B. F.; Geri, J. B.; Oakley, J. V.; Fadeyi, O. O.; et al. Small molecule photocatalysis enables drug target identification via energy transfer. *Proc Natl Acad Sci U S A* **2022**, *119* (34), e2208077119.
(9) Geri, J. B.; Oakley, J. V.; Reyes-Robles, T.; Wang, T.; McCarver, S. J.; White, C. H.; Rodriguez-Rivera, F. P.; Parker, D. L., Jr.; Hett, E. C.; Fadeyi, O. O.; et al. Microenvironment mapping via Dexter energy transfer on immune cells. *Science* **2020**, *367* (6482), 1091-1097.
(10) Oakley, J. V.; Buksh, B. F.; Fernandez, D. F.; Oblinsky, D. G.; Seath, C. P.; Geri, J. B.; Scholes, G. D.; MacMillan, D. W. C. Radius measurement via super-resolution microscopy enables the development of a variable radii proximity labeling platform. *Proc Natl Acad Sci U S A* **2022**, *119* (32), e2203027119.
